# Electroencephalography in Subjective Cognitive Decline and Mild Cognitive Impairment: Systematic Review of Biomarkers, Classification, and Prognostic Evidence

**DOI:** 10.2196/92709

**Published:** 2026-09-30

**Authors:** Sifei Lu, Sheng Hu, Junxuan Huang, Yehua Shi, Dee Yu, Wenting Cao, Mingzhang Yin, Jindong Ding Petersen, Binwen Huang

**Affiliations:** 1 Key Laboratory of Tropical Translational Medicine of Ministry of Education, Department of Epidemiology, School of Public Health, Hainan Medical University, Haikou, Hainan, 571199, China, 86 13256406001; 2 Department of Neurology, The Second Affiliated Hospital of Hainan Medical University, Haikou, China; 3 Library, Hainan Medical University, Haikou, Hainan, China; 4 Department of Public Health, Research Unit for General Practice, University of Copenhagen, Copenhagen, Denmark; 5 Big Data Research Center, School of Intelligent Medicine and Technology, Hainan Medical University, No. 3 Xue Yuan Lu Road, Longhua District, Haikou, Hainan, 571199, China, 86 18976660867; 6 School of Software, Northwestern Polytechnical University, Xi' an, Shaanxi, China

**Keywords:** electroencephalography, mild cognitive impairment, subjective cognitive decline, event-related potential, Alzheimer disease

## Abstract

**Background:**

Subjective cognitive decline (SCD) and mild cognitive impairment (MCI) are heterogeneous clinical states that may represent early or at-risk stages of Alzheimer disease (AD) and other dementias in some individuals. Improved characterization and risk stratification in these populations may facilitate timely evaluation and intervention. Electroencephalography (EEG), a noninvasive, cost-effective neurophysiological technique with high temporal resolution, holds significant potential for elucidating neural mechanisms and providing candidate neurophysiological markers associated with SCD and MCI.

**Objective:**

The study aims to systematically synthesize evidence on group-level EEG biomarkers, EEG-based classification models, the evaluation of EEG in screening or diagnostic pathways, and EEG-based prediction of progression in SCD and MCI.

**Methods:**

A search was conducted across PubMed, Web of Science, Cochrane Library, MEDLINE (via Ovid), Scopus, Wanfang Data, CQVIP, Yiigle, and CNKI databases to include reports published in English or Chinese, which reported group-level EEG differences, EEG-based classification, screening or diagnostic evaluations, or prognostic outcomes in SCD and MCI. A total of 2 independent reviewers screened the titles and abstracts. Prediction-model reports were assessed using PROBAST+AI (Prediction Model Risk of Bias Assessment Tool and Applicability Assessment for AI), prognostic-factor reports using QUIPS (Quality in Prognosis Studies), and other observational reports using design-specific Joanna Briggs Institute checklists. Certainty of evidence was assessed using the GRADE (Grading of Recommendations Assessment, Development, and Evaluation) system.

**Results:**

A total of 20 reports were included, representing a maximum of 3853 confirmed-deduplicated participants after correction of a nested subsample and removal of confirmed duplicate participants across reports, of whom 2927 diagnosed with SCD/MCI/AD or other dementias. Of these, 10 reports were assessed as prediction or AI model reports, 9 as nondiagnostic observational reports, and 1 as a prognostic-factor study. Although potentially informative between-group EEG differences and preliminary model performance were reported, no conventional diagnostic test-accuracy study was identified. The certainty of evidence was very low across all 4 evidence bodies.

**Conclusions:**

The available evidence suggests that EEG is a promising noninvasive modality for characterizing neurophysiological alterations associated with SCD and MCI. EEG-based classification and prognostic models have also shown encouraging preliminary performance. However, the current evidence is more supportive of biomarker discovery and model development than of established clinical screening or diagnosis. Translation into routine practice will require prospective reports with standardized EEG procedures, representative clinical populations, prespecified thresholds, participant-level data separation, and independent external validation. These findings provide a basis for evaluating EEG as a potential adjunctive or triage tool in future clinical pathways.

## Introduction

Subjective cognitive decline (SCD) and mild cognitive impairment (MCI) are heterogeneous clinical states that may precede dementia in some individuals but do not inevitably progress to dementia [1-4]. MCI is characterized by objective cognitive impairment without functional loss sufficient for a diagnosis of dementia [1], whereas SCD refers to a self-experienced persistent decline, despite performance within the normal range on standardized cognitive tests [2]. Both conditions are associated with heterogeneous underlying causes and variable risks of subsequent cognitive decline [2-4].

About 57 million people worldwide are affected by dementia and more than 60% in low- and middle-income nations. Global population aging will push prevalence to 78 million by 2030 and 139 million by 2050, creating a major socioeconomic strain on public health. In 2019, global dementia-related societal costs reached US $1.3 trillion and were forecast to surpass US $2.8 trillion by 2030 amid growing caseloads and higher care costs [5]. China, undergoing rapid demographic aging, currently has 15 million adults aged 60 years and older with dementia and 40 million with MCI, some of whom are at increased, but heterogeneous, risk of progression to dementia [6]. The 2024 Lancet Commission estimated that addressing 14 modifiable risk factors could prevent or delay approximately 45% of dementia cases [7]. Although antiamyloid therapies, such as lecanemab, can modestly slow decline in selected patients with biomarker-confirmed early Alzheimer disease (AD), their costs, monitoring requirements, and potential adverse effects restrict population-level accessibility [8,9]. These considerations reinforce the importance of scalable approaches for early detection and intervention [10]. The assessment and etiological characterization of MCI remain challenging. Clinicians typically rely on neuropsychological instruments such as the Mini-Mental State Examination and Montreal Cognitive Assessment. Brief cognitive screening instruments are influenced by educational, linguistic, and cultural factors and do not independently establish the underlying cause of cognitive impairment [11,12]. Biomarker-based assessment using magnetic resonance imaging (MRI), amyloid positron emission tomography (PET), or cerebrospinal fluid can provide important etiological information, but availability, cost, procedural burden, and invasiveness may limit their use in population-level screening [13,14].

Electroencephalography (EEG) records neural electrical activity with millisecond temporal resolution and is relatively inexpensive, portable, and suitable for repeated assessment. Changes in spectral power, neural oscillatory slowing, functional connectivity, signal complexity, and other nonlinear properties have been reported in SCD and MCI [15-17]. Machine-learning methods may further combine multidimensional EEG features into individualized classification or prediction models. Nevertheless, differences in participant selection, diagnostic definitions, recording paradigms, preprocessing pipelines, feature calculation, decision thresholds, and validation strategies have produced heterogeneous findings and impeded clinical translation.

Previous reviews have provided important but narrower syntheses. Ehteshamzad [18] primarily examined resting-state EEG or EEG-based AD diagnosis and progression. Paitel et al [19,20] focused on cognitive event–related potentials in MCI and AD; Ulbl and Rakusa [21] and Pérez et al [16] primarily focused on EEG findings in SCD; and Aviles et al [22] and Acharya et al [23] emphasized machine- or deep-learning classification of MCI and AD. In contrast, the present review jointly evaluates SCD and MCI as adjacent populations for biomarker discovery and EEG-based model-development research, compares conventional EEG biomarkers with EEG-based prediction models, and incorporates evidence from both international and Chinese databases. It also links multiple publications derived from shared cohorts and examines methodological limitations relevant to diagnostic performance, reproducibility, and clinical feasibility. This review addressed four prespecified but analytically distinct questions: (1) whether EEG features differed between participants with SCD, MCI, AD, and healthy controls (HC); (2) whether EEG-based statistical or AI models could classify these groups; (3) whether EEG had been evaluated as a screening or diagnostic test within an intended clinical pathway; and (4) whether baseline EEG features or EEG-based models could predict progression to dementia. EEG may provide candidate neurophysiological markers for characterizing SCD and MCI, but its clinical screening and diagnostic performance remain uncertain.

## Methods

### Eligibility Criteria

This review followed PRISMA (Preferred Reporting Items for Systematic Reviews and Meta-Analyses) 2020 guidelines (Checklist 1) [24]. We included original reports that clearly defined SCD or MCI, enrolled a relevant sample with a reported mean or median age of 60 years or older, used EEG or event-related potentials, and reported extractable between-group, classification, screening or diagnostic, and prognostic outcomes. For mixed-age populations, separately reported eligible subgroup data were used. We excluded animal reports, unrelated populations, severe-dementia-only reports, nonoriginal publications, duplicate reports without additional relevant data, and reports lacking relevant EEG outcomes or sufficient methodological information.

### Information Sources and Search Strategies

We searched PubMed, Web of Science, MEDLINE via Ovid, Scopus, the Cochrane Library, CNKI, Wanfang Data, CQVIP, and Yiigle from inception to September 25, 2025, and updated the search on August 22, 2026. Controlled vocabulary and free-text terms related to cognitive impairment, SCD, and EEG were used. Full search strategies are provided in Tables S1 to S9 in Multimedia Appendix 1. The strategy was developed by SL, checked by JH, and supervised by a librarian (MY). Only full-text papers published in English or Chinese were eligible.

### Selection Process and Data Extraction

After duplicate removal in EndNote (Clarivate Analytics), 2 reviewers (SL and JH) independently screened titles and abstracts and assessed full-text reports. Disagreements were resolved by consensus or adjudication by a third reviewer (SH). Reasons for full-text exclusion were recorded, and reference lists of included reports were manually searched.

Data were independently extracted in duplicate using a piloted standardized form. Extracted variables included study design, participants, diagnostic definitions and reference standards, EEG acquisition and features, outcomes, comparisons, and effect or performance estimates. For prediction-model reports, we additionally extracted development, validation, and test samples; participant-level vs epoch-level splitting; cross-validation; feature selection; hyperparameter tuning; class-imbalance handling; calibration; external validation; and possible data leakage. Missing information was recorded as “not reported,” and study authors were not contacted. No automation tools were used.

When multiple models were reported, we prioritized external validation, followed by participant-level held-out testing and internal cross-validation. At the same validation level, the prespecified primary or final model, or the most completely reported model, was selected. Reports from overlapping cohorts were retained as separate reports but were not counted as independent participant samples.

### Synthesis Methods

Evidence was organized into 4 prespecified bodies: between-group EEG differences, EEG-based classification, EEG as a screening or diagnostic test, and prediction of progression to dementia. Results were synthesized narratively because of substantial heterogeneity in populations, EEG methods, reference standards, outcomes, and validation procedures. Consequently, no meta-analysis, subgroup analysis, meta-regression, or sensitivity analysis was performed.

### Quality Assessment of the Included Reports

Two reviewers independently assessed risk of bias, with disagreements resolved by consensus or adjudication by SH. Prediction and AI model reports were assessed using PROBAST+AI (Prediction Model Risk of Bias Assessment Tool and Applicability Assessment for AI) [25], nonmodel prognostic-factor reports using QUIPS (Quality in Prognosis Studies), and other observational reports using the corresponding Joanna Briggs Institute (JBI) checklist [26-29]. Because these tools assess different methodological constructs, their findings were reported separately and were not converted into a common cross-tool quality or risk-of-bias scale. PROBAST+AI and QUIPS overall judgments were reported according to the guidance for the respective tools. JBI checklist findings were reported at the item level without numerical scoring or author-defined overall categories. Domain-level PROBAST+AI judgments with study-specific rationales, item-level JBI findings, and domain-level QUIPS judgments are presented in Tables S10A-S14 in Multimedia Appendix 1.

### Certainty and Reporting-Bias Assessment

Two reviewers independently assessed certainty for each of the 4 evidence bodies. Between-group and progression evidence was assessed using GRADE (Grading of Recommendations Assessment, Development, and Evaluation) for observational evidence, whereas classification and screening or diagnostic evidence was assessed using adapted GRADE-diagnostic test accuracy principles [30,31]. Certainty was rated as high, moderate, low, or very low according to risk of bias, inconsistency, indirectness, imprecision, and publication or reporting bias.

Because the classification tasks and performance measures were heterogeneous, certainty concerned the reproducibility and applicability of out-of-sample model performance rather than pooled diagnostic accuracy. No conventional diagnostic test-accuracy study was identified; therefore, the PET-referenced model-development study was treated as indirect screening evidence.

Reporting bias was assessed qualitatively for each evidence body by considering protocol availability, suspected missing results, multiplicity of EEG features and models, and language and publication-status restrictions. Funnel plots and small-study-effect tests were not used because no comparable meta-analysis was performed and each evidence body contained fewer than 10 sufficiently comparable reports.

## Results

### Study Characteristics

The paper selection process is presented in Figure 1. Among the 20 included reports, 10 models of prediction or AI models were assessed using PROBAST+AI, 9 nondiagnostic observational reports using design-specific JBI checklists, and 1 prognostic-factor report using QUIPS. Because these tools assess different methodological constructs, their findings were not combined into a single cross-tool risk-of-bias category. Tool-specific assessments are summarized below and reported in detail in Tables S10A to S14 in Multimedia Appendix 1.

**Figure 1.**
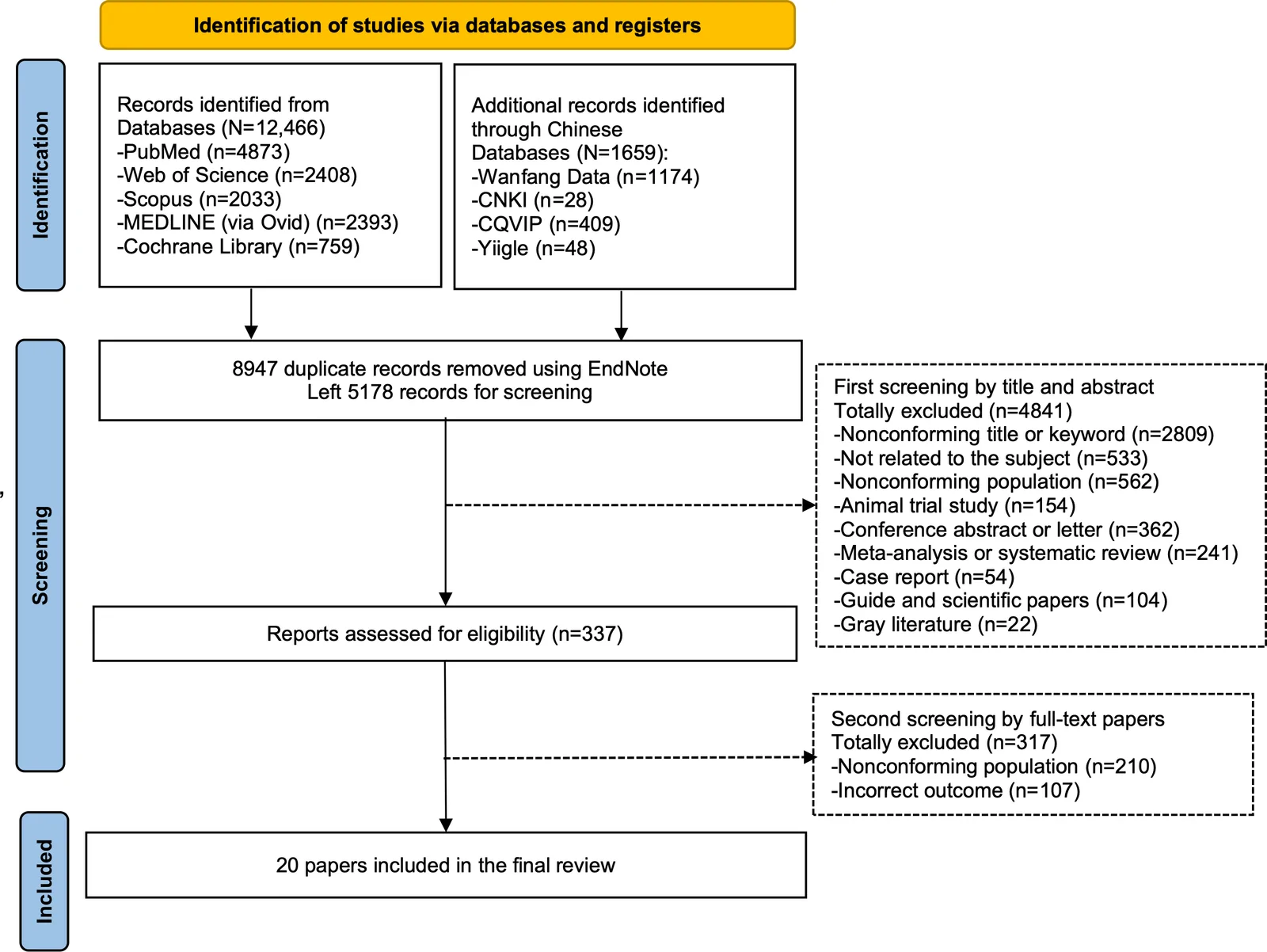
PRISMA (Preferred Reporting Items for Systematic Reviews and Meta-Analyses) flow diagram of the search strategy.

The corrected sum of report-level sample sizes was 3954. After the 101 participants shared by the 2 Sibilano reports were counted once, the confirmed-deduplicated upper bound was 3853 participants: 1039 with SCD/subjective cognitive impairment (SCI), 876 with MCI, 887 with AD or dementia, and 125 with other dementias, 926 cognitively unimpaired controls. The main characteristics of each included study are presented in Table 1.

**Table 1.** Study and demographic information of all included reports.

Study	Design	Participants, n	Sample characteristics, n (mean, SD or median, IQR)	Female/male, n	Contribution to synthesis	Reference-standard note
Lazarou et al [32] (2020)	Cross-sectional	92	20 (73.20, SD 8.17) AD^a^, 30 (70.40, SD 5.96) MCI^b^, 20 (64.90, SD 7.92) SCD^c^, 22 (67.22, SD 4.03) HC^d^	61/31	Between-group EEG^e^ differences	Clinical diagnosis based on established criteria, neuropsychological assessment, MRI^f^, and laboratory work-up
Gouw et al [33] (2017)	Longitudinal	205	142 (68.30, SD 7.40) MCI, 63 (66.20, SD 8.20) SCD	103/102	Prediction of progression	SCD/MCI by standard criteria and amyloid positivity based primarily on CSF^g^ Aβ42
Sibilano et al [34] (2023)	Cross-sectional underlying cohort	118	45 (74.26, SD 8.20) MCI, 56 (66.26, SD 8.72) SCD, 17 (64.29, SD 4.77) HC	75/43	EEG-based classification	SCD-I/NIA-AA^h^ clinical and neuropsychological classification
Sibilano et al [35] (2024)	Cross-sectional same 101 participants, unique contribution =0	101	45 (74.26, SD 8.20) MCI, 56 (66.26, SD 8.72) SCD	68/33	EEG-based classification	SCD-I/NIA-AA classification
Kim et al [36] (2023)	Cross-sectional	311	54 (74.50, SD 6.1) MCI (+), 61 (71.50, SD 6.8) MCI (−), 36 (72.00, SD 5.9) SCD (+), 160 (71.30, SD 6.9) SCD (−)	98/145 missing, n=68	Screening for amyloid pathology	18F-florbetaben PET^i^ visual read was the reference standard
Lazarou et al [37] (2022)	Longitudinal	73	12 (70.50, SD 7.69) AD, 23 (69.61, SD 6.91) MCI, 17 (66.82, SD 8.20) SCD, 21 (62.62, SD 13.86) HC	48/25	Between-group EEG differences; exploratory progression	Neuropsychiatrist diagnosis using established criteria and comprehensive clinical work-up
Ganapathi et al [38] (2022)	Cross-sectional	161	qEEG^j^/ERP^k^, 39 (74.30, SD 8.00) dementia, 53 (72.80, SD 6.7) MCI, 69 (70.60, SD 6.5) SCI	90/71	EEG-based classification	Consensus diagnosis by 2 dementia specialists
Briels et al [39] (2020)	Cross-sectional (2 cohorts)	809	Cohort 1: 214 (67.00, SD 8) AD, 197 (62.00, SD 8) SCD; Cohort 2: 196 (69.00, SD 10) AD, 202 (60.00, SD 10) SCD	363/446	Between-group EEG differences	biomarker-defined A/T/N subgroup available
Huggins et al [40] (2021)	Cross-sectional	141	52 (82.30, SD 4.70) AD, 37(78.40, SD 5.10) MCI, 52 (79.60, SD 6.00) HA^l^	—^m^	EEG-based classification	Clinical NINCDS^n^/NIA-AA/Petersen-type labels; epoch
Engedal et al [41] (2020)	Longitudinal	200	88 (71.20, SD 8.10) MCI, 45 (68.00, SD 8.00) SCD, 67 (65.60, SD 7.30) HC	105/95	Prediction of progression	Baseline and follow-up diagnoses assigned by experienced physicians independently of qEEG
Cave et al [42] (2025)	Cross-sectional	28	14 (67.9, SD 5.3) SCI, 14 (68.2, SD 5.2) HCs	16/12	Between-group EEG differences	Group definition relied on subjective concern and MoCA^o^ thresholds
Jiang et al [43] (2025)	Cross-sectional	41	21 (70.33, SD 2.85) MCI, 20 (70.00, SD 3.39) NC^p^	27/14	EEG-based classification/preliminary screening	2018 Chinese MCI guideline and cognitive/functional assessment
Jeong et al [44] (2021)	Cross-sectional	46	23 (72.14, SD 3.19) SCD, 23 (72.02, SD 3.13) controls	35/11	Between-group EEG differences	Neurologist classification using clinical and neuropsychological criteria
Zawiślak-Fornagiel et al [45] (2024)	Cross-sectional	53	30 (70.00, SD 6.38) aMCI^q^, 23 (66.00, SD 6.50) CogN^r^	—	Between-group EEG differences	Memory-clinic clinical/neuropsychological diagnosis
Kim et al [46] (2024)	Cross-sectional	58	10 (71.20, SD 6.76) AD, 18 (66.17, SD 8.74) aMCI, 10 (69.60, SD 3.47) naMCI^s^, 20 (63.42, SD 11.18) SCD	30/28	EEG-based classification	Clinical/SNSB-II^t^ and CDR^u^ labels
Shim et al [47] (2022)	Cross-sectional	95	26 (73.81, SD 5.59) SCD amyloid PET (+), 69 (72.55, SD 4.69) SCD amyloid PET (−)	53/42	Between-group EEG differences; indirect screening evidence	18F-florbetaben PET visual read used to define amyloid status
Jiao et al [48] (2023)	Cross-sectional	890	330 (64.6, SD 9.75) AD, 57 (67.18, SD 9.80) VCI^v^, 47 (61.36, SD 8.69) FTD^w^, 21 (72.01, SD 9.07) DLB^x^, 189 (64.77, SD 9.30) MCI, 246 (63.85, SD 8.20) HC	—	EEG-based classification	Established syndrome-specific clinical criteria; non-AD dementias contributed only to specified differential comparisons
Katayama et al [49] (2023)	Cross-sectional	449	47 (73, IQR 69-78) MCI, 402 (73, IQR 70-78) HC	249/200	EEG-based classification	MCI required ≥1 domain at least 1.5 SD below norms with preserved ADL^y^
Mazzon et al [50] (2018)	Cross-sectional	26	11 (76.72, SD 4.98) MCI, 8 (74.63, SD 5.73) SCI, 7 (74.28, SD 5.15) CS^z^	16/10	Between-group EEG differences	MCI followed 2011 NIA-AA criteria; SCI required subjective complaints without objective impairment
Lazarou et al [51] (2018)	Cross-sectional	57	14 (72.3, SD 6.54) AD, 17 (68.7, SD 6.19) MCI, 14 (69.9, SD 6.83) SCI, 12 (65.8, SD 2.82) HC	38/19	Between-group EEG differences	Neuropsychiatrist diagnosis using established criteria and full clinical work-up

a AD: Alzheimer disease.

b MCI: mild cognitive impairment.

c SCD: subjective cognitive decline.

d HC: healthy control.

e EEG: electroencephalography.

f MRI: magnetic resonance imaging.

g CSF: cerebrospinal fluid.

h NIA-AA: National Institute on Aging–Alzheimer’s Association.

i PET: positron emission tomography.

j qEEG: quantitative electroencephalography.

k ERP: event-related potential.

l HA: healthy adults.

m Not available.

n NINCDS: National Institute of Neurological and Communicative Disorders and Stroke.

o MoCA: Montreal Cognitive Assessment.

p NC: normal control.

q aMCI: amnestic mild cognitive impairment.

r CogN: cognitive normal.

s naMCI: nonamnestic mild cognitive impairment.

t SNSB-II: Seoul Neuropsychological Screening Battery–II.

u CDR: Clinical Dementia Rating.

v VLC: vascular cognitive impairment.

w FTD: frontotemporal dementia.

x DLB: dementia with Lewy bodies.

y ADL: activities of daily living.

z CS: cognitive stimulation.

### Differences in EEG Features Between Groups

Study-level findings and principal limitations across the 4 prespecified evidence bodies are summarized in Table 2. Nine reports examined group-level differences in spectral power, functional connectivity, signal complexity, or event-related potentials among participants with SCD, MCI, AD, other dementias, and cognitively unimpaired controls [32,37,39,42,44,45,47,50,51]. Across the spectral reports, cognitive impairment was generally associated with greater low-frequency activity and reduced higher-frequency activity. Jeong et al [44] reported a partial increase in frontal delta power and a decrease in occipital alpha-1 power in participants with SCD compared with community controls. Zawislak-Fornagiel et al [45] similarly found lower global relative beta power and higher theta and delta power in participants with amnestic MCI than in cognitively unimpaired participants. Among participants with SCD, Shim et al [47] found that participants with amyloid PET–positive had higher delta and lower alpha-1 activity than participants with amyloid PET–negative. Mazzon et al [50] also identified differences in alpha-band activity and EEG complexity among participants with SCI, MCI, and controls, particularly during a memory task.

Connectivity and task-related reports also identified group-level differences, although the specific measures and directions varied. Lazarou et al [32,37] found differences in clustering coefficient, network strength, and other connectome measures across SCD, MCI, AD, and control groups during resting-state and visual memory paradigms. Participants with SCD generally showed intermediate network organization between cognitively unimpaired controls and participants with MCI or AD. Briels et al [39] reported that functional connectivity measures differed significantly across various diagnostic groups in dimensions sensitive to changes associated with AD pathology. Task-related differences were also reported for the N170 event-related potential across SCI, MCI, AD, and control groups [51]. Cave et al [42] assessed the differences in resting-state EEG between older adults with SCI and HCs, revealing distinct neurophysiological signatures despite normal objective cognitive function, thus highlighting the potential of EEG-derived features for research-stage characterization of SCD.

In summary, EEG measures, including connectivity features and machine learning–based approaches, have shown exploratory potential for differentiating SCD from MCI.

**Table 2.** Summary of findings according to the prespecified review questions.

Evidence body and included report	Population/comparison or task	Key finding	Interpretation and principal limitation
Differences in EEG features between groups			
Briels et al [39] (2020)	SCD^a^ vs AD^b^ across 2 cohorts	AD showed reduced alpha/beta amplitude-envelope correlation with leakage correction; theta connectivity findings varied by metric	Reproducibility differed across connectivity measures; not a diagnostic-accuracy study
Cave et al [42] (2025)	SCI^c^ vs HC^d^; eyes-open/eyes-closed EEG	Frequency-component amplitudes differed according to group, sex, anxiety, and depressive symptoms	Small case-control sample; group assignment relied on self-report/MoCA^e^ and does not establish screening accuracy
Jeong et al [44] (2021)	SCD vs NC^f^	SCD showed localized frontal delta increase and occipital alpha-1 decrease	No consistent source-level or connectivity differences; no multiplicity-adjusted diagnostic model
Zawiślak-Fornagiel et al [45] (2024)	aMCI^g^ vs cognitively normal controls	aMCI showed lower global beta power, higher theta/delta power, and reduced right centroparietal coherence	Case-control design; multiple comparisons and transportability remain concerns
Shim et al [47] (2022)	Amyloid-positive vs amyloid-negative SCD	Amyloid-positive SCD showed increased delta and reduced alpha-1 activity	Association with PET^h^ status; no prespecified EEG^i^ threshold or patient-level accuracy evaluation
Mazzon et al [50] (2018)	SCI, MCI^j^, and controls during rest/memorization	Frontal alpha attenuation and reduced EEG complexity were reported in SCI, with more diffuse changes in MCI	Very small exploratory sample; findings should be described as candidate features
Lazarou et al [51] (2018)	HC, SCI, MCI, and AD during facial processing	N170 amplitude/topography differed across cognitive groups	Exploratory ERP^k^ association; no independent validation or clinical diagnostic threshold
Lazarou et al [32] (2020)	HC, SCD, MCI, and AD	SCD showed intermediate graph-theory network organization between HC and MCI/AD	Cross-sectional association; possible cohort overlap with other Lazarou reports
Lazarou et al [37] (2022)	HC, SCD, MCI, and AD during visual-memory/attention tasks	SCD showed intermediate network disorganization during encoding and retrieval	Small longitudinal cohort; possible overlap and multiplicity concerns
EEG-based classification			
Huggins et al [40] (2021)	AD vs MCI vs healthy aging	AlexNet mean accuracy 98.9% (SD 0.4%)	Images/epochs were randomly split; participant-level independence was not reported, creating high leakage risk
Jiang et al [43] (2025)	MCI vs NC using reduced electrode configurations	Derivation AUC=0.683‐0.830; external OCL4 AUC=0.771, sensitivity 0.918, specificity 0.610	External evaluation included only 10 participants and CIs were not reported
Kim et al [46] (2024)	SCD vs naMCI^l^ vs aMCI vs AD	Best encoding-state cKNN^m^: accuracy 93.10%, *F*_1_ 92.78%, K=0.816; class AUCs^n^=0.890‐1.000	Feature selection was not clearly nested within leave-one-subject-out validation
Jiao et al [48] (2023)	HC vs MCI; HC vs AD; HC vs MCI vs AD	LDA^o^ accuracy 79.8% and *F*_1_ 80.9% for HC vs MCI; approximately 85.8% for HC vs AD and approximately 70% for 3 classes	Participant-level separation was not clearly reported for classification; possible epoch-level leakage
EEG as a screening or diagnostic test			
Katayama et al [49] (2023)	Community MCI vs HC	Best SVM^p^: accuracy 0.7929, sensitivity 0.7163, specificity 0.8632, AUC=0.8495	Feature construction was not clearly repeated within every resampling loop
Sibilano et al [34] (2023)	SCD vs MCI; HC vs SCD vs MCI	Delta transformer: epoch accuracy 67.4%, AUC=0.807; participant accuracy 76.2%. Three-class participant accuracy was approximately 54%	No external validation
Sibilano et al [35] (2024)	SCD vs MCI	Best transformer: epoch accuracy 65.4% (95% CI 63.7%‐67.1%); participant accuracy 65.7%	Repeated comparison of configurations on one holdout; same cohort as Sibilano et al [34] (2023)
Ganapathi et al [38] (2022)	SCD vs MCI vs dementia	qEEG^q^/ERP model AUC=0.79; qEEG/ERP plus volumetric MRI^r^ AUC=0.87	Feature screening/stepwise selection was not clearly nested within leave-one-out validation
Kim et al [36] (2023)	Amyloid PET positivity in SCD/MCI	Holdout sensitivity 90.9%, specificity 76.7%, accuracy 82.9%, AUC=0.84	The random holdout was used for final model/cutoff selection and was not true external validation
Prediction of progression to dementia			
Gouw et al [33] (2017)	Amyloid-positive SCD/MCI followed for clinical progression	In SCD, higher delta/theta and lower alpha/peak frequency were associated with faster progression	Prognostic-factor associations; no validated individual-level prediction model
Engedal et al [41] (2020)	Baseline SCD/MCI to dementia over approximately 5 years	Dementia Index AUC=0.78 (95% CI 0.70‐0.85), sensitivity 71%, specificity 68%‐69%, accuracy 69%	Cutoff selected in the evaluation cohort; incremental models lacked separate validation
Lazarou et al [37] (2022)	SCD conversion over 3 years	Lower baseline network measures were reported among SCD participants who progressed to MCI	Small exploratory subgroup and possible cohort overlap; not a validated prognostic model

a SCD: subjective cognitive decline.

b AD: Alzheimer disease.

c SCI: subjective cognitive impairment.

d HC: healthy control.

e MoCA: Montreal Cognitive Assessment.

f NC: normal control.

g aMCI: amnestic mild cognitive impairment.

h PET: positron emission tomography.

i EEG: electroencephalography.

j MCI: mild cognitive impairment.

k ERP: event-related potential.

l naMCI: nonamnestic mild cognitive impairment.

m cKNN: constrained k-nearest neighbor.

n AUC: area under the receiver operating characteristic curve.

o LDA: linear discriminant analysis.

p SVM: support vector machine.

q qEEG: quantitative electroencephalography.

r MRI: magnetic resonance imaging.

### EEG-Based Classification Models

Eight reports developed or evaluated statistical, machine-learning, or deep-learning models for classifying cognitive-status groups [34,35,40,43,46,48,50,51]. The classification tasks varied substantially and included binary discrimination between SCD and MCI, discrimination between MCI and cognitively unimpaired controls, and multiclass classification of SCD, MCI, AD, other dementias, and healthy aging. Predictor sets included spectral power, connectivity, event-related potentials, electrode-specific measures, cognitive-task EEG features, and combinations of EEG with demographic, cognitive, MRI, or biochemical information.

Sibilano et al [34,35] evaluated transformer-based models using the same underlying cohort of participants with SCD and MCI. The delta-band data produced the best performance for SCD vs MCI classification, whereas alpha- and theta-band data contributed to the 3-class classification of HC, SCD, and MCI. Because the 2023 and 2024 reports analyzed overlapping participants, their findings were treated as 2 analyses of one cohort rather than as independent validation reports. Huggins et al [40] reported a mean accuracy of 98.9% (SD 0.4%) for 3-class classification of AD, MCI, and healthy aging using an AlexNet-based deep-learning approach. However, the participant-level independence of the image-based data split was insufficiently documented, and no external validation was reported.

Jiang et al [43] evaluated electrode configurations for classifying MCI and controls and reported sensitivity of up to 96.2% for a 4-electrode occipital configuration, although specificity was substantially lower for some configurations, indicating a risk of false-positive classification. Kim et al [46] also presented findings that linked EEG features in resting and memory-encoding states to cognitive performance across different dementia stages, supporting further evaluation of EEG-derived features as candidate biomarkers rather than established tools for early detection. Other reports combined EEG features with cognitive, MRI, demographic, or biochemical variables to classify MCI, AD, or other dementia categories [38,48,49].

All contributing classification-model reports were judged to be at high risk of bias. Common limitations included small effective sample sizes relative to model complexity, inadequate separation of feature selection or hyperparameter tuning from performance evaluation, possible participant- or epoch-level data leakage, incomplete reporting of calibration, and a lack of independent external validation. A concise summary of the target tasks, validation designs, key performance estimates, and principal limitations is presented in Table 3; full model-development and validation details are provided in Table S17 in Multimedia Appendix 1.

**Table 3.** Development, validation, and performance summary of the included electroencephalography (EEG) prediction and AI models.

Included report	Model-study type	Target outcome	Development sample	Validation strategy	Key performance	External validation	Main risk to interpretation
Huggins et al [40] (2021)	Development	AD^a^/MCI^b^/healthy-aging classification	141 participants; 16,197 epoch images	Random 10-fold image-level split (8/10 train, 1/10 validation, 1/10 test)	Accuracy 98.9% (SD 0.4%)	No	Probable participant leakage across folds
Engedal et al [41] (2020)	Development and evaluation	Conversion to dementia	Existing DI^c^ plus new incremental models	Evaluation in 200 participants with follow-up; no resampling for new models	AUC^d^ 0.78; sensitivity 71%; specificity 68%‐69%; accuracy 69%	Evaluation of preexisting DI, but cutoff fitted in the current cohort	Cutoff selection and incremental model fitting in evaluation data
Jiang et al [43] (2025)	Development and evaluation	MCI vs NC^e^	41 participants	Apparent derivation plus external sample of 10	External AUC 0.771; sensitivity 0.918; specificity 0.610	Yes, n=10	Very imprecise external performance; derivation feature screening
Kim et al [46] (2024)	Development	Four-class AD spectrum	58 participants	Leave-one-subject-out cross-validation	Accuracy 93.10%; *F*_1_ 92.78%	No	Feature selection not clearly nested; small classes
Jiao et al [48] (2023)	Development	HC^f^/MCI/AD classification	Classification sample: HC 246, MCI 189, AD 330	Random 80/20 samples; 5-fold validation in training set	HC vs MCI accuracy 79.8%; 3-class approximately 70%	No	Possible epoch-level leakage and data-driven feature selection
Katayama et al [49] (2023)	Development	MCI vs HC	449 participants	Monte Carlo 5-fold cross-validation, 100 iterations	Best AUC 0.8495	No	Feature construction not clearly nested; ROSE^g^ resampling
Sibilano et al [35] (2024)	Development	SCD^h^ vs MCI	101 participants	20% participant holdout plus 5-fold development validation	Epoch accuracy 65.4%; participant accuracy 65.7%	No	Repeated holdout use; overlaps as Sibilano et al [34] (2023)
Sibilano et al [34] (2023)	Development	SCD/MCI and HC/SCD/MCI	118 participants	Leave-one-subject-out cross-validation	SCD vs MCI AUC 0.807; participant accuracy 76.2%	No	Validation subset incompletely described; overlaps as Sibilano et al [35] (2024)
Kim et al [36] (2023)	Development	Amyloid PET^i^ positivity	235 development; 76 random holdout	5-fold development CV^j^ plus internal holdout	Holdout AUC 0.84; sensitivity 90.9%; specificity 76.7%	No true external validation	Holdout reused for final model/cutoff selection after extensive model search
Ganapathi et al [38] (2022)	Development	SCD/MCI/dementia classification	161 qEEG^k^/ERP^l^; 111 multimodal	Leave-one-subject-out cross-validation	AUC 0.79; multimodal AUC 0.87	No	Variable screening and stepwise selection not clearly nested

a AD: Alzheimer disease.

b MCI: mild cognitive impairment.

c DI: dementia index.

d AUC: area under the receiver operating characteristic curve.

e NC: normal control.

f HC: healthy control.

g ROSE: Random Over-Sampling Examples.

h SCD*:* subjective cognitive decline.

i PET: positron emission tomography.

j CV: cross-validation.

k EEG: quantitative electroencephalography.

l ERP: event-related potential.

### EEG as a Screening or Diagnostic Test

No included report met all criteria for a conventional diagnostic test-accuracy study. Specifically, no study evaluated a prespecified and fixed EEG test or threshold against an independent reference standard in a representative clinical screening population while also defining whether EEG was intended as a replacement, add-on, or triage test. Consequently, sensitivity and specificity were not pooled.

One PET-referenced machine-learning report provided indirect evidence relevant to this question. Kim et al developed an EEG-based model to identify amyloid PET positivity among 311 participants with SCD or MCI. The study reported a sensitivity of up to approximately 90% in its model evaluation. However, the analysis remained a model-development study: the final model and threshold had not been prospectively fixed, model selection was conducted among multiple candidate approaches, independent external validation was absent, and the intended position of EEG in a clinical diagnostic pathway was not specified. The results, therefore, did not establish the accuracy of a deployable EEG screening test [36].

Shim et al [47] also compared quantitative EEG features between amyloid PET-positive and amyloid PET-negative participants with SCD. However, this report assessed group-level biomarker associations and did not evaluate a prespecified diagnostic threshold or provide a complete participant-level diagnostic-accuracy analysis. It was therefore synthesized with the group-difference evidence rather than as a diagnostic test-accuracy study.

Accordingly, the available evidence was insufficient to determine whether EEG could be used as a replacement, an add-on, or a triage test for SCD, MCI, AD, or underlying amyloid pathology.

### Prediction of Progression to Dementia

Three longitudinal reports examined associations between baseline EEG measures and subsequent progression to dementia [33,37,41]. Gouw et al [33] investigated participants with amyloid-positive SCD or MCI who did not have dementia at baseline and found that lower alpha peak frequency and higher delta or theta activity were associated with faster clinical progression. However, these findings represent exploratory group-level associations rather than evidence of individual-level prognostic prediction. Across the 3 studies, sample sizes were limited, and none developed and externally validated a multivariable prediction model or reported model calibration. The certainty of this evidence was rated as very low, and the available findings are insufficient to support individualized risk estimation or clinical decision-making.

Engedal et al [41] evaluated an automated quantitative EEG dementia index for predicting conversion among participants with SCD or MCI over approximately 5 years. The index showed moderate discrimination, with reported sensitivity of 71%, specificity of 68%, accuracy of 69%, and an area under the curve of approximately 0.78 in the reported analyses. The preexisting dementia index was evaluated in a new cohort; however, the operating cutoff was selected using the evaluation data. Newly fitted incremental models received only apparent evaluation, and calibration was not reported.

Lazarou et al [37] examined longitudinal changes or progression in relation to baseline high-density EEG network characteristics across preclinical and clinical stages of cognitive impairment. The study provided evidence that altered network organization may be associated with subsequent cognitive deterioration, but the sample was small and no validated individual-level risk prediction model was developed.

The prognostic findings were limited by small sample sizes, differences in the definition of progression, heterogeneous follow-up periods, incomplete adjustment for confounding, and the absence of independent external validation. Thus, baseline EEG may contain prognostic information, but the magnitude, reproducibility, and clinical applicability of this information remain uncertain.

### Risk of Bias and Methodological Quality

Of the 20 included reports, 10 reports on prediction or AI models were assessed using PROBAST+AI, 9 nondiagnostic observational reports using design-specific JBI checklists, and 1 prognostic-factor report using QUIPS. All 10 model-development assessments were judged to be at high risk of bias. The analysis domain was the predominant source of bias. The main concerns included small effective sample sizes, data-driven feature or model selection, inadequate separation of model development from performance evaluation, possible participant- or epoch-level data leakage, limited calibration assessment, and the absence of independent external validation. The separate model-evaluation components in Engedal et al [41] and Jiang et al [43] were also judged to be at high risk of bias, primarily because of data-dependent cutoff selection and the very small external evaluation sample, respectively.

For the 9 nonmodel observational reports assessed using JBI checklists, findings were reported at the checklist-item level rather than converted into overall low-, moderate-, or high-risk categories. Among the 5 analytical cross-sectional reports, Briels et al [39] and Mazzon et al [50] met all 8 checklist criteria. Shim et al [47] met 7 criteria, with 1 item rated as unclear. Jeong et al [44] and Lazarou et al [32] had concerns mainly related to the identification or management of confounding and the appropriateness of the statistical analysis. The 3 case-control reports had item-level concerns involving group comparability or matching, control of confounding, and statistical analysis. The cohort report had concerns regarding confounding management, completeness of follow-up, and statistical analysis. No composite overall JBI rating was assigned to these reports.

The prognostic-factor report assessed using the QUIPS tool was judged to be at a high overall risk of bias, driven principally by high attrition and additional concerns regarding study participation, confounding, and statistical analysis or reporting (Table 4). Tool-specific judgments and study-specific rationales are presented in Tables S10A to S14 in Multimedia Appendix 1.

**Table 4.** Tool-specific summary of methodological quality and risk-of-bias assessments^a^.

Assessment framework	Reports, n	Reports assessed	Tool-specific findings	Principal methodological concerns
PROBAST+AI^b^: model development	10	Huggins et al [40] (2021); Engedal et al [41] (2020); Jiang et al [43] (2025); Kim et al [46] (2024); Jiao et al [48] (2023); Katayama et al [49] (2023); Sibilano et al [35] (2024); Sibilano et al [34] (2023); Kim et al [36] (2023); Ganapathi et al [38] (2022)	All 10 model-development assessments were judged to be at high risk of bias	Small or selected samples; limited effective sample sizes; data-driven feature or model selection; unclear participant-level independence; possible data leakage; limited calibration assessment; and no independent external validation
PROBAST+AI: model evaluation	2	Engedal et al [41] (2020); Jiang et al [43] (2025)	Both evaluation components were judged to be at high risk of bias. These components were part of the included reports and were not counted as additional reports	Engedal et al [41] selected the cutoff using the evaluation cohort. Jiang et al [43] used an external sample of only 10 participants and did not report precision estimates
JBI^c^ analytical cross-sectional checklist	5	Briels et al [39] (2020); Jeong et al [44] (2021); Shim et al [47] (2022); Mazzon et al [50] (2018); Lazarou et al [32] (2020)	Two reports met all 8 criteria; 1 met 7 criteria, with 1 item rated as unclear; and 2 had concerns involving specific checklist items. No composite overall rating was assigned	Concerns mainly involved the identification or management of confounding and the appropriateness of statistical analysis
JBI case-control checklist	3	Cave et al [42] (2025); Zawiślak-Fornagiel et al [45] (2024); Lazarou et al [51] (2018)	Item-level findings were reported without a composite overall rating	Concerns involved group comparability or matching, confounding control, and statistical analysis
JBI cohort checklist	1	Lazarou et al [37] (2022)	Item-level findings were reported without a composite overall rating	Concerns involved confounding management, completeness of follow-up, and statistical analysis
QUIPS^d^	1	Gouw et al [33] (2017)	The report was judged to be at high overall risk of bias	High attrition risk and additional concerns regarding study participation, confounding, and statistical analysis or reporting

a Because these tools assess different methodological constructs, their findings were not converted into a common numerical score or cross-tool risk-of-bias category. JBI findings were reported at the checklist-item level without numerical scoring or an author-defined composite overall rating. The 2 PROBAST+AI model-evaluation components were contained within the included reports and were not counted as additional reports.

b PROBAST+AI: Prediction Model Risk of Bias Assessment Tool+AI.

c JBI: Joanna Briggs Institute.

d QUIPS: Quality in Prognosis Studies.

### Certainty of the Evidence and Reporting Bias

The certainty of the evidence was rated as very low for all 4 prespecified outcome bodies (Table 5). Evidence concerning between-group EEG differences was downgraded because of risk of bias, substantial clinical and methodological inconsistency, indirectness to individual-level diagnosis, imprecision, and suspected reporting bias. Classification evidence was limited by high risk of bias in model development, heterogeneous classification tasks and algorithms, reliance on internal validation, possible data leakage, and limited precision. Evidence concerning EEG as a screening or diagnostic test was based on only 1 indirect, PET-referenced, model-development report and did not establish the accuracy of a deployable EEG test within an intended clinical pathway. Prognostic evidence was limited by small samples, heterogeneous definitions of progression, incomplete control of confounding, and the absence of externally validated and calibrated prediction models.

**Table 5.** Certainty of evidence and synthesis-level reporting-bias assessment.

Prespecified outcome body	Contributing evidence, n	Framework	Risk of bias	Inconsistency	Indirectness	Imprecision	Reporting/publication bias	Overall certainty	Interpretation
Differences in EEG^a^ features between groups	9 reports; independent cohort count uncertain because of possible Lazarou overlap	GRADE^b^ for observational evidence	Serious	Very serious	Serious	Serious	Strongly suspected	Very low	The direction and anatomical distribution of EEG differences were not sufficiently consistent for a clinically transferable biomarker conclusion
EEG-based classification of SCD^c^, MCI^d^, AD^e^, and cognitively unimpaired controls	7 underlying cohorts (8 reports)	Adapted GRADE-DTA^f^ principles for model performance	Very serious	Serious	Serious	Serious	Strongly suspected	Very low	Reported classification performance was limited by small samples, heterogeneous tasks/models, internal validation, and possible data leakage
EEG as a screening or diagnostic test	1 report	GRADE-DTA	Very serious	Not assessable	Very serious	Very serious	Strongly suspected	Very low	Only one PET^g^-referenced model study contributed indirect development evidence; clinical-pathway accuracy remains uncertain
Prediction of progression to dementia	3 reports	GRADE for observational/prognostic evidence	Very serious	Serious	Serious	Serious	Strongly suspected	Very low	Associations were suggestive, but no externally validated, calibrated prognostic model was identified

a EEG: electroencephalography.

b GRADE: Grading of Recommendations Assessment, Development, and Evaluation.

c SCD: subjective cognitive decline.

d MCI: mild cognitive impairment.

e AD: Alzheimer disease.

f DTA: diagnostic test accuracy.

g PET: positron emission tomography.

Reporting bias was strongly suspected for all 4 evidence bodies. Most reports had no publicly available protocol or prespecified analysis plan, and many evaluated multiple EEG features, thresholds, comparisons, or candidate models, increasing the possibility of selective reporting. The restriction to English- and Chinese-language full-text publications and the exclusion of gray literature may also have contributed to missing evidence. Funnel plots and statistical tests for small-study effects were not performed because no meta-analysis using a common effect measure was conducted and fewer than 10 sufficiently comparable reports contributed to each evidence body. Detailed GRADE judgments and downgrade rationales are presented in Table S15 in Multimedia Appendix 1.

## Discussion

### Principal Findings

This systematic review synthesized evidence across 4 related but distinct questions: between-group differences in EEG features, EEG-based classification of cognitive status, the use of EEG as a screening or diagnostic test, and the prediction of progression to dementia. Several reports described potentially informative group-level EEG differences and preliminary classification performance in MCI and SCD; however, these findings do not establish individual-level screening or diagnostic accuracy [52].

Several studies reported potentially informative differences in spectral power, functional connectivity, event-related potentials, and other quantitative EEG measures. Nevertheless, the direction and magnitude of these findings were not fully consistent. Differences in recording conditions, electrode configurations, frequency-band definitions, cognitive tasks, diagnostic criteria, and adjustment for potential confounders may have contributed to this heterogeneity. In addition, many reports examined multiple EEG features without clearly identifying a prespecified primary feature or threshold, which may have increased the possibility of selective reporting.

There were 8 reports that developed or evaluated EEG-based models for classifying individuals with SCD, MCI, AD, or cognitively unimpaired controls. Although several studies reported encouraging discrimination or classification performance, most estimates were derived from relatively small and selected samples and were primarily based on apparent performance or internal validation. All contributing classification-model reports were judged to be at high risk of bias. These judgments largely reflected limited effective sample sizes, nonnested feature selection or hyperparameter tuning, potential participant-level or epoch-level information leakage, reuse of test data during model selection, limited calibration assessment, and scarce independent external validation [53]. These factors may have resulted in the overestimation of model performance. The findings should therefore be considered preliminary evidence of technical feasibility that requires further validation before clinical implementation.

None of the included reports evaluated a prespecified EEG test using all elements typically expected in a conventional diagnostic test-accuracy study, including a representative clinical population, an appropriate reference standard, and a clearly defined clinical pathway. The PET-referenced machine-learning study provided potentially valuable evidence regarding amyloid-status classification, although its relevance to the clinical diagnosis of SCD, MCI, or AD was indirect. Longitudinal studies also suggested that selected EEG measures may be associated with subsequent cognitive progression, but evidence supporting a calibrated and independently validated individual-risk model remains limited. Overall, the certainty of evidence was rated as very low for all 4 prespecified outcomes, indicating that the estimates and interpretations may change as more rigorous evidence becomes available [54].

### Research Trends and Future Directions

Emerging trends in EEG research related to MCI and SCD are being significantly influenced by advancements in deep-learning models, multimodal neuroimaging techniques, and candidate biomarkers intended to support research-stage characterization and prognostic evaluation across the AD continuum.

Recent studies have reported preliminary performance of deep-learning approaches in analyzing resting-state EEG signals to classify individuals at different stages of cognitive decline. For instance, Sibilano et al used a transformer architecture with self-attention mechanisms, which provided potentially interpretable attention patterns for the model’s decisions, enabling discrimination between SCD and MCI with notable accuracy. Additionally, their work highlighted how attention scores could facilitate a better understanding of the EEG patterns indicative of cognitive decline [34,35]. Sibilano et al [34,35] further built on this by implementing a deep-learning model that classified participants with SCD and MCI, showing encouraging but internally validated performance through multiple frequency bands of EEG and employing an innovative approach with multihead attention.

Moreover, Jiao et al [48] have highlighted the need for a holistic view, combining EEG metrics with demographic and biochemical data (such as cerebrospinal fluid and apolipoprotein E genotype) to enhance predictive capabilities regarding AD progression. Their findings indicated that EEG-based classifications showed higher performance in the reported comparisons in terms of accuracy, especially in differentiating between MCI and AD when combined with other biomarkers [35].

Furthermore, the exploration of specific EEG responses to emotional stimuli, as investigated by Lazarou et al [51], revealed that the amplitude of the N170 event-related potential could play a critical role in differentiating cognitive statuses. Their work showed significant variations in brain activation patterns among individuals with SCD, MCI, and HCs during the processing of facial stimuli, indicating the potential utility of such metrics in understanding cognitive decline stages.

Finally, Huggins et al [40] developed a deep-learning model for classifying patients with AD, MCI, and healthy aging using resting-state EEG signals. Their results indicated a high apparent accuracy, demonstrating technical feasibility, although the apparent performance requires participant-independent external validation before any diagnostic interpretation.

In summary, this body of research concerning MCI and SCD is rapidly evolving, driven by innovative neural network architectures, integration of multimodal data, and the exploration of novel biomarkers. This trend points toward a future where EEG-derived features may warrant further evaluation as candidate components of multimodal research and future clinical-assessment pathways.

### Strengths and Limitations

This review has several strengths. First, it organized the evidence according to 4 prespecified questions rather than treating all EEG reports as a single diagnostic evidence base. Second, study selection and data extraction were independently conducted by 2 reviewers using standardized procedures. Third, risk of bias was evaluated using tools matched to the study objective and design: PROBAST+AI for prediction-model reports, QUIPS for prognostic-factor reports, and design-specific JBI checklists for other observational reports. Prediction-model reports were further categorized into development and performance-evaluation components, with signaling-question responses and applicability judgments reported at the model level. Fourth, the certainty of evidence and synthesis-level reporting bias were formally assessed for each outcome body. Reports from confirmed or potentially overlapping cohorts were also identified to reduce duplicate participant counting and overinterpretation of correlated evidence.

This systematic review has several limitations that should be acknowledged. Although 5178 records were initially screened, only 20 studies met the inclusion criteria, which may limit the generalizability of the findings, particularly for underrepresented populations or research methodologies. The search was restricted to English- and Chinese-language publications and predominantly focused on peer-reviewed literature. Studies published in other languages, dissertations, conference proceedings, and unpublished reports may therefore have been missed, increasing the possibility of publication and reporting bias. Finally, most included studies were small, methodologically heterogeneous, and conducted in selected research samples. This review was not prospectively registered, and no publicly accessible protocol was prepared; consequently, independent verification of whether all methodological decisions were prespecified is limited.

### Conclusion

This systematic review suggests that EEG is a promising noninvasive modality for investigating neurophysiological alterations across SCD, MCI, AD, and cognitively unimpaired populations. The included reports identified potentially informative group-level differences in spectral, connectivity, event-related potential, and other quantitative EEG measures. Several EEG-based classification and prognostic models also reported encouraging preliminary performance. Together, these findings support continued investigation of EEG as a candidate biomarker and a model-development platform for early cognitive impairment.

Nevertheless, substantial heterogeneity in study populations, diagnostic definitions, EEG acquisition and preprocessing, feature selection, reference standards, and validation procedures limits the direct clinical interpretation of the available findings. Most model-performance estimates were derived from selected samples and internal validation, and none of the included reports evaluated a fixed EEG test within a representative clinical diagnostic pathway. The certainty of the evidence therefore remains very low. Rather than establishing immediate clinical applicability, the present review defines the methodological steps needed to advance the field. Addressing these methodological priorities may help determine which EEG features and models are sufficiently reproducible and transportable for clinical evaluation. With rigorous prospective validation, EEG may ultimately contribute as an adjunctive or triage tool within multimodal pathways for the assessment of early cognitive impairment.

## Supplementary material

10.2196/92709 Multimedia Appendix 1 Search strategies and quality assessment of selection study.

10.2196/92709 Checklist 1 PRISMA 2020 checklist.

## Abbreviations

AD: Alzheimer disease
EEG: electroencephalography
GRADE: Grading of Recommendations Assessment, Development, and Evaluation
HC: healthy control
JBI: Joanna Briggs Institute
MCI: mild cognitive impairment
MRI: magnetic resonance imaging
PET: positron emission tomography
PRISMA: Preferred Reporting Items for Systematic Reviews and Meta-Analyses
PROBAST+AI: Prediction Model Risk of Bias Assessment Tool and Applicability Assessment for AI
QUIPS: Quality In Prognosis Studies
SCD: subjective cognitive decline
SCI: subjective cognitive impairment
