# Supplementary material for: Electroencephalography in Subjective Cognitive Decline and Mild Cognitive Impairment: Systematic Review of Biomarkers, Classification, and Prognostic Evidence
Source: J Med Internet Res. 2026 Sep 30;28:e92709. doi: 10.2196/92709 (PMC13626404; doi:10.2196/92709)
Supplement: Supplementary file 1 [file jmir-v28-e92709-s001.docx]

Supplementary Material 1. Searching strategy Table S1-S9.

Supplementary Material 2. Quality assessment of selection study Table S10-S18.

| **Supplementary Material 1. Searching strategy Table S01-S09.** | |
| --- | --- |
| **S01 PubMed** | |
| Search date: 25 September 2025; updated on 22 August 2026. | |
| Search | Results |
| ( "Cognitive Dysfunction"[MeSH Terms] OR MCI[Title/Abstract] OR SCD[Title/Abstract] OR "mild cognitive impairment"[Title/Abstract] OR "mild cognitive impairments"[Title/Abstract] OR "cognitive dysfunction"[Title/Abstract] OR "cognitive dysfunctions"[Title/Abstract] OR "cognitive disorder"[Title/Abstract] OR "cognitive disorders"[Title/Abstract] OR "cognitive impairment"[Title/Abstract] OR "cognitive impairments"[Title/Abstract] OR "cognitive decline"[Title/Abstract] OR "mild cognitive decline"[Title/Abstract] OR "mental deterioration"[Title/Abstract] OR "subjective cognitive decline"[Title/Abstract] OR "subjective cognitive impairment"[Title/Abstract] OR "subjective memory complaint"[Title/Abstract] OR "subjective memory complaints"[Title/Abstract] OR "subjective memory decline"[Title/Abstract] ) AND ( "Electroencephalography"[MeSH Terms] OR "Evoked Potentials"[MeSH Terms] OR "Event-Related Potentials, P300"[MeSH Terms] OR EEG[Title/Abstract] OR qEEG[Title/Abstract] OR electroencephalograph*[Title/Abstract] OR "resting state EEG"[Title/Abstract] OR "resting-state EEG"[Title/Abstract] OR "quantitative EEG"[Title/Abstract] OR "background EEG"[Title/Abstract] OR "ongoing EEG"[Title/Abstract] OR "brain rhythm"[Title/Abstract] OR "brain rhythms"[Title/Abstract] OR "EEG phase synchronization"[Title/Abstract] OR "event-related potential"[Title/Abstract] OR "event-related potentials"[Title/Abstract] OR "event related potential"[Title/Abstract] OR "event related potentials"[Title/Abstract] OR ERP[Title/Abstract] OR ERPs[Title/Abstract] OR "evoked potential"[Title/Abstract] OR "evoked potentials"[Title/Abstract] OR P300[Title/Abstract] OR P300b[Title/Abstract] ) AND humans[Filter] AND english[Filter] | 4,873 |

| **S02 Web of Science** | | | | |
| --- | --- | --- | --- | --- |
| Search date: 25 September 2025; updated on 22 August 2026. | | | | |
| # | Search | Database | Editions | Results |
| 1 | TS=(MCI OR "mild cognitive impairment*" OR "mild cognitive dysfunction" OR "mild cognitive disorder*" OR "mild cognitive decline" OR "subjective cognitive decline" OR "subjective cognitive impairment" OR "subjective memory complaint*" OR "subjective memory decline" OR (SCD NEAR/3 cognit*)) AND TS=(EEG OR qEEG OR electroencephalo* OR "resting state EEG" OR "ongoing EEG" OR "background EEG" OR "quantitative EEG" OR "EEG phase synchron*" OR "event-related potential*" OR ERP OR ERPs OR "evoked potential*" OR P300*) | Web of Science Core Collection | Science Citation Index Expanded  (SCI-EXPANDED)--1900-present  Social Sciences Citation Index  (SSCI)--2014-present | 2408 |

| **S03 Cochrane Library** | | |
| --- | --- | --- |
| Search date: 25 September 2025; updated on 22 August 2026. | | |
| Comment: | |  |
| ID | Search | Hits |
| #1 | MeSH descriptor: [Cognitive Dysfunction] explode all trees | 5545 |
| #2 | MeSH descriptor: [Electroencephalography] explode all trees | 7343 |
| #3 | MeSH descriptor: [Evoked Potentials] explode all trees | 4501 |
| #4 | (MCI OR SCD OR "Cognitive Dysfunction" OR "Cognitive Dysfunctions" OR "Cognitive Disorder" OR "Cognitive Disorders" OR "Cognitive Impairment" OR "Cognitive Impairments" OR "Cognitive Decline" OR "Mental Deterioration"):ti,ab,kw | 29708 |
| #5 | (EEG OR qEEG OR Electroencephalography OR Electroencephalographic OR Electroencephalogram OR Electroencephalograms OR Electrocorticography OR "brain rhythm" OR "brain rhythms" OR "event-related potential" OR "event-related potentials" OR "event related potential" OR "event related potentials" OR ERP OR ERPs OR "evoked potential" OR "evoked potentials" OR P300 OR P300b):ti,ab,kw | 27265 |
| #6 | #1 OR #4 | 29744 |
| #7 | #2 OR #3 OR #5 | 27631 |
| #8 | #6 AND #7 | 1530 |
| #9 | #8 AND English:la | 1501 |
| #10 | #9 AND journal:pt | 759 |

| **S04 MEDLINE (via Ovid)** | | |
| --- | --- | --- |
| Search date: 25 September 2025; updated on 22 August 2026. | | |
| # ▲ | Searches | Results |
| 1 | Cognitive Dysfunction/ | 55839 |
| 2 | exp Electroencephalography/ | 189521 |
| 3 | exp Evoked Potentials/ | 132332 |
| 4 | (MCI or (SCD adj3 cognit*) or mild cognitive impair* or mild cognitive dysfunction* or mild cognitive disorder* or mild cognitive decline* or subjective cognitive decline or subjective cognitive impair* or subjective memory complaint* or subjective memory decline). ti,ab,kw. | 47137 |
| 5 | (EEG or qEEG or electroencephalo* or resting state EEG or resting-state EEG or quantitative EEG or background EEG or ongoing EEG or EEG phase synchron* or event-related potential* or event related potential* or ERP or ERPs or evoked potential* or P300 or P300b). ti,ab,kw | 226036 |
| 6 | 1 or 4 | 83791 |
| 7 | 2 or 3 or 5 | 348127 |
| 8 | 6 and 7 | 2520 |
| 9 | journal article.pt | 38345870 |
| 10 | 8 and 9 | 2492 |
| 11 | exp animals/ not humans.sh. | 5492720 |
| 12 | 10 not 11 | 2393 |

| **S05 Scopus** | |
| --- | --- |
| Search date: 25 September 2025; updated on 22 August 2026. | |
| Searches | Results |
| ( TITLE-ABS-KEY( MCI OR "mild cognitive impairment*" OR "mild cognitive dysfunction" OR "mild cognitive disorder*" OR "mild cognitive decline" OR "subjective cognitive decline" OR "subjective cognitive impairment" OR "subjective memory complaint*" OR "subjective memory decline" OR (SCD W/3 cognit*) ) AND TITLE-ABS-KEY( EEG OR qEEG OR electroencephalo* OR "resting state EEG" OR "ongoing EEG" OR "background EEG" OR "quantitative EEG" OR "EEG phase synchron*" OR "event-related potential*" OR ERP OR ERPs OR "evoked potential*" OR P300* ) ) AND ( LIMIT-TO ( EXACTKEYWORD,"Human" ) ) AND ( LIMIT-TO ( DOCTYPE,"ar" ) ) AND ( LIMIT-TO ( LANGUAGE,"English" ) ) | 2033 |

| **S06 Wan Fang** | | |  |
| --- | --- | --- | --- |
| Search date: 25 September 2025; updated on 22 August 2026. | | | |
| Search number | Query | Search settings/filters | Results |
| 1 | [主题] (脑电图 OR EEG OR 事件相关电位 OR ERP OR P300 OR 诱发电位)  English translation: [Topic] (EEG OR Electroencephalography OR "event-related potential" OR ERP OR P300 OR "evoked potential") | Fuzzy matching; Journal articles; no publication-date restriction; Language: Chinese | 254842 |
| 2 | [主题] (轻度认知障碍 OR MCI OR 主观认知障碍 OR 认知障碍)  English translation: [Topic] (MCI OR "mild cognitive impairment" OR "subjective cognitive impairment" OR "cognitive impairment") | Fuzzy matching; Journal articles; no publication-date restriction; Language: Chinese | 31794 |
| 3 | #1 AND #2 | - | 1174 |

| **S07 CQVIP ((VIP Chinese Science and Technology Journal Database))** | |
| --- | --- |
| Search date: 25 September 2025; updated on 23 August 2026. |  |
| Searches | Results |
| [题名或关键词] (脑电图 OR EEG OR 事件相关电位OR ERP OR P300 OR 诱发电位) AND [题名或关键词] (轻度认知障碍 OR MCI OR 主观认知障碍 OR 认知障碍)  English translation: [Title or Keyword] (EEG OR Electroencephalography OR "event-related potential" OR ERP OR P300 OR "evoked potential") AND [Title or Keyword] (MCI OR "mild cognitive impairment" OR "subjective cognitive impairment" OR "cognitive impairment") | 409 |

| **S08 Yiigle** | |  |
| --- | --- | --- |
| Search date: 25 September 2025; updated on 23 August 2026. | |  |
| Searches | Filters | Results |
| (脑电图 OR EEG OR 事件相关电位 OR ERP OR P300 OR 诱发电位) AND (轻度认知障碍 OR MCI OR 主观认知障碍 OR 认知障碍)  English translation: (EEG OR Electroencephalography OR "event-related potential" OR ERP OR P300 OR "evoked potential") AND ("mild cognitive impairment" OR MCI OR "subjective cognitive impairment" OR "cognitive impairment"). | 文献类型：原创论文；研究类型：基于临床人群研究；研究方法：随机对照试验、干预性研究、队列研究、横断面研究、病例对照研究或描述性研究  Document type: Original Article; Study population: Clinical population study; Study design: Randomized controlled trial, interventional study, cohort study, cross-sectional study, case-control study, or descriptive study | 48 |

| **S09 CNKI** | |
| --- | --- |
| Search date: 25 September 2025; updated on 23 August 2026. | |
| Searches | Results |
| [主题] ('脑电图(eeg)' OR 事件相关电位OR ERP OR P300 ) AND [主题] (轻度认知障碍 OR MCI OR 认知障碍)  English translation: [Topic] ('Electroencephalography(eeg)' OR "event-related potential" OR ERP OR P300) AND [Topic] (MCI OR "mild cognitive impairment" OR "cognitive impairment") | 28 |

| **Supplementary Material 2**  **Table S10A. Domain-level PROBAST+AI judgments for model development** | | | | | | | | | | | |
| --- | --- | --- | --- | --- | --- | --- | --- | --- | --- | --- | --- |
| Author | Year | **Risk of bias** | | | | | **Applicability** | | | | |
|  |  | Participants and data  sources ^a^ | Predictors ^b^ | Outcome ^c^ | Analysis ^d^ | Overall development RoB ^h^ | | Participants and data  sources ^e^ | Predictors ^f^ | Outcome ^g^ | Overall applicability concern ^i^ |
| Huggins et al. | 2021 | H | U | U | H | H | | H | L | L | H |
| Engedal et al. | 2020 | L | L | L | H | H | | L | L | L | L |
| Jiang et al. | 2025 | H | U | U | H | H | | H | L | L | H |
| Kim et al. | 2024 | H | U | U | H | H | | H | L | U | H |
| Jiao et al. | 2023 | H | U | U | H | H | | U | L | U | U |
| Katayama et al. | 2023 | L | U | H | H | H | | L | L | H | L |
| Sibilano et al. | 2024 | H | U | U | H | H | | L | L | L | L |
| Sibilano et al. | 2023 | H | U | U | H | H | | H | L | L | H |
| Kim et al. | 2023 | H | U | L | H | H | | L | L | H | H |
| Ganapathi et al. | 2022 | H | L | L | H | H | | H | L | L | H |
| Judgments are presented at the domain level. Signaling questions were used to inform these judgments but are not reproduced in this table. H indicates high risk of bias or high applicability concern; L, low risk or low concern; and U, unclear risk or concern.  **a. Participants and data sources**  1.1 Did the in- and exclusions of study participants result in a representative dataset?  **b. Predictors**  2.1 Were predictors defined and assessed in a similar way for all participants?  2.2 Was any pre-processing of predictors similar for all participants?  **c. Outcome**  3.1 Were outcomes defined and assessed appropriately?  3.2 Were outcomes defined and assessed in a similar way for all participants?  3.3 Were outcome assessments made without use or knowledge of predictor data?  **d. Analysis**  4.1 Was there evidence that the sample size was reasonable?  4.2 Were continuous and categorical predictors handled appropriately?  4.3 Were participants with missing or censored data handled appropriately in the analysis?  4.4 Were methods used to address potential model overfitting?  **e. Participants and data sources**  Concern that the (data of the) included participants do not match the review question or the assessor’s intended use of the prediction model.  **f. Predictors**  Concern that the definition, pre-processing, assessment, or timing of assessment of the predictors in the model do not match the review question or the assessor’s intended use.  **g. Outcome**  Concern that the outcome, its definition, assessment, or timing of assessment do not match the review question or the assessor’s intended use.  **h. Overall risk of bias**  Low risk: If all four domains were rated low risk of bias.  High risk: If at least one domain was rated high risk of bias.  Unclear risk of bias: If at least one domain was rated unclear and no domain was rated high risk of bias.  **i. Applicability concerns**  Low risk: If all three domains were rated low concern for applicability.  High risk: If at least one domain was rated high concern for applicability.  Unclear: If at least one domain was rated unclear concern for applicability and no domains were rated high concern. | | | | | | | | | | | |

| **Table S10B. Summary domain-level PROBAST+AI judgments for model evaluation** | | | | | | | | | | | |
| --- | --- | --- | --- | --- | --- | --- | --- | --- | --- | --- | --- |
| Author | Year | **Risk of bias** | | | | | **Applicability** | | | | |
|  |  | Participants and data  sources ^a^ | Predictors ^b^ | Outcome ^c^ | Analysis ^d^ | Overall development RoB ^h^ | | Participants and data  sources ^e^ | Predictors ^f^ | Outcome ^g^ | Overall applicability concern ^i^ |
| Engedal et al. | 2020 | L | L | L | H | H | | L | L | L | L |
| Jiang et al. | 2025 | H | U | U | H | H | | H | L | L | H |
| Judgments are presented at the domain level. Signaling questions were used to inform these judgments but are not reproduced in this table. H indicates high risk of bias or high applicability concern; L, low risk or low concern; and U, unclear risk or concern.    **a. Participants and data sources**  1.1 Did the in- and exclusions of study participants result in a representative dataset?  **b. Predictors**  2.1 Were predictors defined and assessed in a similar way for all participants?  2.2 Was any pre-processing of predictors similar for all participants?  **c. Outcome**  3.1 Were outcomes defined and assessed appropriately?  3.2 Were outcomes defined and assessed in a similar way for all participants?  3.3 Were outcome assessments made without use or knowledge of predictor data?  **d. Analysis**  4.1 Was there evidence that the sample size was reasonable?  4.2 Were continuous and categorical predictors handled appropriately?  4.3 Were participants with missing or censored data handled appropriately in the analysis?  4.4 Were methods used to address potential model overfitting?  **e. Participants and data sources**  Concern that the (data of the) included participants do not match the review question or the assessor’s intended use of the prediction model.  **f. Predictors**  Concern that the definition, pre-processing, assessment, or timing of assessment of the predictors in the model do not match the review question or the assessor’s intended use.  **g. Outcome**  Concern that the outcome, its definition, assessment, or timing of assessment do not match the review question or the assessor’s intended use.  **h. Overall risk of bias**  Low risk: If all four domains were rated low risk of bias.  High risk: If at least one domain was rated high risk of bias.  Unclear: If at least one domain was rated unclear risk of bias and no domains were rated high concern.  **i. Applicability concerns**  Low risk: If all three domains were rated low concern for applicability.  High risk: If at least one domain was rated high concern for applicability.  Unclear: If at least one domain was rated unclear concern for applicability and no domains were rated high concern.  The quality of the included studies was assessed using the Joanna Briggs Institute (JBI) critical appraisal tool. In the assessment table, “Y” stands for “Yes”, “N” for “No”, and “U” for “Unclear”. The tool includes the following eight questions: 1) Were the criteria for inclusion in the sample clearly defined? 2) Were the study subjects and the setting described in detail? 3) Was the exposure measured in a valid and reliable way? 4) Were objective, standard criteria used for measurement of the condition? 5) Were confounding factors identified? 6) Were strategies to deal with confounding factors stated? 7) Were the outcomes measured in a valid and reliable way? 8) Was appropriate statistical analysis used? | | | | | | | | | | | |

| **S11 JBI critical appraisal checklist of analytical cross-sectional studies.** | | | | | | | | | |
| --- | --- | --- | --- | --- | --- | --- | --- | --- | --- |
| Study | JBI’s critical appraisal questions | | | | | | | | Item-level summary |
|  | Q1 | Q2 | Q3 | Q4 | Q5 | Q6 | Q7 | Q8 |  |
| Briels et al., 2020 | Y | Y | Y | Y | Y | Y | Y | Y | All 8 criteria were met. |
| Jeong et al., 2021 | Y | Y | Y | Y | N | N | Y | N | Concerns regarding identification and management of confounding and statistical analysis (Q5, Q6, and Q8). |
| Shim et al., 2022 | Y | Y | Y | Y | Y | Y | Y | U | Seven criteria were met; the appropriateness of the statistical analysis was unclear (Q8). |
| Mazzon et al. | Y | Y | Y | Y | Y | Y | Y | Y | All 8 criteria were met. |
| Lazarou et al., 2020 | Y | Y | Y | Y | Y | N | Y | N | Concerns regarding strategies for managing confounding and statistical analysis (Q6 and Q8). |

Note. Y=yes; N=no; U=unclear; NA=not applicable. Q1: Were the criteria for inclusion in the sample clearly defined? Q2: Were the study participants and setting described in detail? Q3: Was the exposure measured validly and reliably? Q4: Were objective, standard criteria used to measure the condition? Q5: Were confounding factors identified? Q6: Were strategies to address confounding stated? Q7: Were outcomes measured validly and reliably? Q8: Was appropriate statistical analysis used? Responses are reported at the item level. No numerical quality score or overall low-, moderate-, or high-risk category was calculated because the checklist does not prescribe a validated weighting scheme or cutoff for such conversion.

| **S12 JBI critical appraisal checklist for Case-Control Studies Checklist** | | | | | | | | | | | |
| --- | --- | --- | --- | --- | --- | --- | --- | --- | --- | --- | --- |
| **Report** | **Q1** | **Q2** | **Q3** | **Q4** | **Q5** | **Q6** | **Q7** | **Q8** | **Q9** | **Q10** | **Item-level summary** |
| Cave et al., 2025 | Y | Y | Y | Y | Y | Y | Y | Y | NA | N | Most criteria were met; the exposure-period item was not applicable and the statistical analysis raised concern (Q10). |
| Zawiślak-Fornagiel et al., 2024 | U | Y | Y | Y | Y | Y | N | Y | NA | N | Group comparability was unclear; no strategy for dealing with confounding was reported; statistical analysis raised concern (Q1, Q7, and Q10). |
| Lazarou et al., 2018 | N | N | Y | Y | Y | Y | N | Y | NA | U | Groups were not comparable or appropriately matched; no strategy for dealing with confounding was reported; statistical analysis was unclear (Q1, Q2, Q7, and Q10). |

Note. Y=yes; N=no; U=unclear; NA=not applicable. Q1: Were the groups comparable other than the presence of disease in cases or absence of disease in controls? Q2: Were cases and controls matched appropriately? Q3: Were the same criteria used to identify cases and controls? Q4: Was exposure measured in a standard, valid, and reliable way? Q5: Was exposure measured in the same way for cases and controls? Q6: Were confounding factors identified? Q7: Were strategies to deal with confounding stated? Q8: Were outcomes assessed in a standard, valid, and reliable way for cases and controls? Q9: Was the exposure period of interest long enough to be meaningful? Q10: Was appropriate statistical analysis used? Responses are reported at the item level; no numerical score or composite overall category was calculated.

| **S13 Quality assessment results of included studies using JBI critical appraisal checklist for Cohort Studies Tool** | | | | | | | | | | | | |
| --- | --- | --- | --- | --- | --- | --- | --- | --- | --- | --- | --- | --- |
| **Report** | **Q1** | **Q2** | **Q3** | **Q4** | **Q5** | **Q6** | **Q7** | **Q8** | **Q9** | **Q10** | **Q11** | **Item-level summary** |
| Lazarou et al., 2022 | Y | Y | Y | Y | N | Y | Y | Y | U | U | N | Concerns were identified for management of confounding and statistical analysis; completeness of follow-up and strategies for incomplete follow-up were unclear (Q5, Q9-Q11). |

Note. Y=yes; N=no; U=unclear; NA=not applicable. Q1: Were the 2 groups similar and recruited from the same population? Q2: Were exposures measured similarly to assign participants to exposed and unexposed groups? Q3: Was exposure measured validly and reliably? Q4: Were confounding factors identified? Q5: Were strategies to address confounding stated? Q6: Were participants free of the outcome at the start of the study? Q7: Were outcomes measured validly and reliably? Q8: Was follow-up reported and sufficient for outcomes to occur? Q9: Was follow-up complete, and if not, were reasons for loss to follow-up described and explored? Q10: Were strategies used to address incomplete follow-up? Q11: Was appropriate statistical analysis used? Responses are reported at the item level; no numerical score or composite overall category was calculated.

| **S14 QUIPS risk-of-bias assessment of prognostic-factor studies** | | | | | | | |
| --- | --- | --- | --- | --- | --- | --- | --- |
| **Report** | **Study participation** | **Study attrition** | **Prognostic factor measurement** | **Outcome measurement** | **Study confounding** | **Statistical analysis/reporting** | **Overall RoB** |
| Gouw et al., 2017 | Moderate | High | Low | Low | Moderate | Moderate | **High** |

Note. RoB=risk of bias. Overall RoB was judged high because study attrition was high and concerns also remained in study participation, confounding, and statistical analysis/reporting. Prognostic-factor and outcome measurement were judged low risk.

| **S15 Certainty-of-evidence assessment for the four evidence bodies** | | | | | | | | | |
| --- | --- | --- | --- | --- | --- | --- | --- | --- | --- |
| **Outcome** | **Independent studies (reports), n** | **Framework** | **Initial certainty** | **Risk of bias** | **Inconsistency** | **Indirectness** | **Imprecision** | **Publication bias** | **Overall certainty** |
| Differences in EEG features between groups | 9 | GRADE for observational evidence | Low | Seriousᵃ | Very seriousᵇ | Seriousᶜ | Seriousᵈ | Strongly suspectedᵉ | **Very low** |
| EEG-based classification of SCD, MCI, AD, and cognitively unimpaired controls | **7 underlying cohorts (8 reports)** | Adapted GRADE-DTA principles for model performance | High | Very seriousᶠ | Seriousᵍ | Seriousʰ | Seriousⁱ | Strongly suspectedʲ | **Very low** |
| EEG as a screening or diagnostic test | 1 | GRADE-DTA | High | Very seriousᵏ | Not assessableˡ | Very seriousᵐ | Very seriousⁿ | Strongly suspectedᵒ | **Very low** |
| Prediction of progression to dementia | 3 | GRADE for observational/prognostic evidence | Low | Very seriousᵖ | Serious^q^ | Seriousʳ | Seriousˢ | Strongly suspectedᵗ | **Very low** |

| **S16 EEG parameters of all included publications** | | | | | | | |
| --- | --- | --- | --- | --- | --- | --- | --- |
| **Study** | **Year** | **Filters (Hz)** | **EEG outcome measures** | **Sampling Rates (Hz)** | **Impedance (kOhm)** | **Eye condition** | **Reference** |
| Lazarou et al. | 2020 | 5th-order bandpass Butterworth IIR filter (0.3-75) | Pearson Correlation Coefficien; Clustering Coefficient;Strength; Betweenness Centrality | 250 | 50 | closed | Cz |
| Gouw et al. | 2017 | low pass filter 70 Hz or 100 Hz and no notch filter | delta, theta, alpha, beta, gamma and peak frequency | 200 or 250 | <5 | closed | Cz |
| Sibilano et al. | 2023 | A) adapted and employed the PREP pipeline B) 50 Hz notch filter was applied to ensure line noise cleaning | delta, theta, alpha and beta | 512 | <5 | closed and open | n/a |
| Sibilano et al. | 2024 | Bandpass filter (1-30) | delta, theta, alpha and beta | 512 | n/a | closed and open | n/a |
| Heon Kim et al. | 2023 | Bandpass (0.1–45.5) | delta, theta, alpha 1, alpha 2,beta 1, beta 2, beta 3 and gamma | 200 or 250 | 10 | closed | ear |
| Lazarou et al. | 2022 | 5th-order bandpass Butterworth IIR filter (0.3-70) | Pearson Correlation Coefficien; Clustering Coefficient;Strength; Betweenness Centrality | 250 | 50 | Eye-Tracker | Cz |
| Ganapathi et al. | 2022 | n/a | three qEEG/ERP predictors —— P300b amplitude, Reaction Time, and Commission Errors | n/a | n/a | Eyes Open (5-min), Eyes Closed (5-min), Go-No Go button-press task (5-min) | n/a |
| Briels et al. | 2020 | low pass filter ≤ 70 Hz, high pass filter ≥ 0.5 Hz, and no notch filter; and the average reference was used. | Coh, iCoh, PLV, AEC, AEC-c, PLI and wPLI were estimated in five frequency bands: broadband, delta, theta, alpha and beta | 500 | <5 | closed | n/a |

| **S16 (continued)** | | | | | | | |
| --- | --- | --- | --- | --- | --- | --- | --- |
| **Study** | **Year** | **Filters (Hz)** | **EEG outcome measures** | **Sampling Rates (Hz)** | **Impedance (kOhm)** | **Eye condition** | **Reference** |
| Huggins et al. | 2021 | A) using 1–60 Hz band-pass, FIR filter with an order of 330 B) de-noised using independent component analysis and notch filters at 21 and 42 Hz | n/a | 200 | n/a | closed | n/a |
| Engedal et al. | 2020 | applying a 8th-order Butterworth band-pass filter with the chosen band (0.1-70) | delta,theta,alpha and beta | n/a | n/a | closed | n/a |
| Cave et al. | 2025 | acquired DC-70 Hz with a 50 Hz notch filter | delta,theta,alpha and beta | 1000 | <10 | 2 min eyes-open, 2 min eyes-closed, then a 2 min eyes-open | M1 |
| Yi Jiang et al. | 2025 | band-pass filtering was applied to remove unwanted high-frequency noise and low-frequency interference (1-30) | delta,alpha | 250 | <10 | focus on computer screen | ear |
| Jeong et al. | 2021 | analog-filtered with a band pass (0.5-70) | delta, theta, alpha1, alpha2, beta1, beta2, beta3, and gamma | 200 | <5 | open (30 s) closed(30 s), 10 times | ear |
| Fornagiel et al. | 2024 | A) band-pass filter with cutoff frequencies (0.5-70) B) initially filtered with 50 Hz notch filter C) The qEEG parameters were extracted based on PSD computed with the multitaper method from 0.5 to 45 Hz. | delta,theta,alpha,beta and gamma | 128 | n/a | closed | n/a |

| **S16 (continued)** | | | | | | | |
| --- | --- | --- | --- | --- | --- | --- | --- |
| **Study** | **Year** | **Filters (Hz)** | **EEG outcome measures** | **Sampling Rates (Hz)** | **Impedance (kOhm)** | **Eye condition** | **Reference** |
| Kim et al. | 2024 | infinite impulse response notch filter (60) | delta band, theta band,alpha band,low beta band,high beta band and gamma band | 512 | <10 | Open (3min) and closed (3min) | n/a |
| Shim et al. | 2022 | n/a | delta, theta, alpha 1, alpha 2, beta 1, beta 2, beta 3, and gamma band;theta-to-alpha, delta-to-alpha, and theta-to-beta | 200 or 250 | <10 | closed | ear |
| Jiao, B et al. | 2023 | band-pass filter (1-55) and further filtered by a 50-Hz notch filter | delta, theta, alpha, beta, and gamma band | 200 | n/a | closed (10min) | n/a |
| Katayama et al. | 2023 | Bandpass filtering (0.53-120) | 1)the loading values for each IC output using the ICA algorithm;2) the peak latency of N2 and P3 and the amplitude | 500 | <10 | closed (5min), fixated on target | ear |
| Mazzon et al. | 2018 | Second order,Butterworth filter (0.5 -60) | delta,theta,alpha1,alpha2, beta1, beta2,gamma;β exponent,fractal dimension | 1024 | n/a | n/a | n/a |
| Lazarou et al. | 2018 | A.)5th-order bandpass butterworth IIR filter (0.1–30) B.) segmented using task trigger timestamps and an epoch time of 1s | N170 component's amplitude latency | 250 | <50 | open | Cz |

AEC amplitude envelope correlation; AEC-c AEC with leakage correction; Coh Mean global coherence; ERP event-related potential; n/a not applicable; iCoh imaginary coherence; PLI phase lag index; PLV phase locking value; PSD power spectral density; wPLI weighted phase lag index

| **Table S17. Diagnostic definitions and reference standards used in the included reports** | | | | | |
| --- | --- | --- | --- | --- | --- |
| **Included report** | **Diagnostic groups / target condition** | **Operational diagnostic definition** | **Reference standard and adjudication** | **Independence from EEG index test** | **Contribution to this review** |
| Briels et al., 2020 | SCD and probable AD dementia; biomarker-defined A/T/N subgroup | SCD was a clinical diagnosis in people with cognitive complaints who did not meet criteria for MCI or dementia. Probable AD dementia was diagnosed according to contemporary clinical criteria. A stricter subgroup contrasted SCD A−T− with AD A+T+. | Multidisciplinary memory-clinic consensus based on clinical assessment, neuropsychological testing, and MRI/CT; CSF amyloid/tau or amyloid PET was incorporated when available and was required only for the A/T/N subgroup. | EEG was not used to establish the clinical diagnosis. | Differences in EEG features between groups. |
| Huggins et al., 2021 | AD, MCI, and healthy ageing (HA) | AD and HA followed NINCDS/NINCDS-ADRDA source-dataset criteria (typical AD: MMSE ≤24, CDR 0.5–2, and functional/cognitive decline during the preceding 12 months; HA: MMSE ≥25 and CDR 0). MCI required objective impairment in ≥1 cognitive domain with preserved functional independence, consistent with Albert/Petersen criteria. | Clinical diagnoses inherited from the Brazilian Behavioural and Cognitive Neurology dataset; no biomarker confirmation was required. | EEG was not described as part of the reference diagnosis. | EEG-based classification models. |

| **Table S17. (continued)** | | | | | |
| --- | --- | --- | --- | --- | --- |
| **Included report** | **Diagnostic groups / target condition** | **Operational diagnostic definition** | **Reference standard and adjudication** | **Independence from EEG index test** | **Contribution to this review** |
| Engedal et al., 2020 | Baseline SCD or MCI; conversion to all-cause dementia and dementia subtypes | MCI followed Winblad et al.; SCD was defined in line with Jessen et al. as subjective decline without MCI or dementia. Follow-up diagnoses included AD (McKhann/NIA-AA), vascular dementia (NINDS-AIREN), DLB, FTD, Parkinson disease dementia, and other specified dementias. | At least 2 experienced physicians assigned baseline and follow-up diagnoses using standardized clinical and cognitive assessments; follow-up dementia diagnosis was made independently of qEEG. | Explicitly independent of qEEG/Dementia Index. | Prediction of progression to dementia. |
| Cave et al., 2025 | SCI and healthy controls | SCI required a self-perceived cognitive decline, an affirmative response to ≥1 cognitive-concern question, and MoCA ≥23. Controls reported no cognitive concern and had MoCA >26; participants self-reported no MCI or dementia. | Group assignment was based on self-report and MoCA eligibility rather than an independent specialist or biomarker reference standard. | EEG did not define groups, but the reference procedure was limited. | Differences in EEG features between groups. |
| Jiang et al., 2025 | MCI and NC | MCI followed the 2018 Chinese guideline: subjective complaint, education-adjusted MoCA <26 and MMSE >24, preserved basic activities (ADL=100) with limited instrumental impairment, and age 65–75 years. NC had no complaint, MoCA >26, MMSE >24, and no major neurologic/psychiatric disorder. | Guideline-based clinical and cognitive classification; no independent biomarker reference standard was reported. | EEG was not used to assign the source labels. | EEG-based classification/screening model. |

| **Table S17. (continued)** | | | | | |
| --- | --- | --- | --- | --- | --- |
| **Included report** | **Diagnostic groups / target condition** | **Operational diagnostic definition** | **Reference standard and adjudication** | **Independence from EEG index test** | **Contribution to this review** |
| Jeong et al., 2021 | SCD and normal controls | Controls met Christensen normal-elderly criteria and cognitive/functional cutoffs (K-MMSE ≥−1 SD, K-IADL ≤0.42, KDSQ ≤6). SCD required a memory complaint with performance within the normal range on the Seoul Neuropsychological Screening Battery and absence of dementia under DSM-IV. | A neurologist assigned diagnoses using clinical and neuropsychological evaluation; no biomarker standard was reported. | EEG was not part of the diagnostic determination. | Differences in EEG features between groups. |
| Zawiślak-Fornagiel et al., 2024 | aMCI and cognitively normal controls | aMCI was identified in routine memory-clinic assessment using clinical and neuropsychological criteria (MMSE 24–27, or MMSE 28–30 with an abnormal Clock Drawing Test or impaired Benton/extended neuropsychological performance). Controls had MMSE 27–30 and Clock Drawing Test 9–10. Nonamnestic MCI, dementia, and secondary causes were excluded. | Clinical interview/informant history, neurologic examination, neuropsychological battery, structural imaging, and laboratory work-up; no amyloid/tau requirement. | EEG was not reported as part of the case definition. | Differences in EEG features between groups. |
| Seul-Kee Kim et al., 2024 | SCD, nonamnestic MCI, amnestic MCI, and AD | All participants underwent SNSB-II. CDR 0 was categorized as SCD, CDR 0.5 as MCI, and CDR 1 as AD. Within MCI, memory performance <−1 SD relative to age/education norms defined aMCI; otherwise participants were categorized as naMCI. | Clinical labels supplied by the recruiting memory-clinic cohort using neuropsychological assessment and CDR; no biomarker confirmation was required. | EEG was not used to establish the class labels. | EEG-based classification models. |

| **Table S17. (continued)** | | | | | |
| --- | --- | --- | --- | --- | --- |
| **Included report** | **Diagnostic groups / target condition** | **Operational diagnostic definition** | **Reference standard and adjudication** | **Independence from EEG index test** | **Contribution to this review** |
| Shim et al., 2022 | High-risk SCD, stratified by cerebral amyloid status | SCD participants had subjective decline without MCI or dementia and objective test scores generally ≥−1.5 SD; the analyzed high-risk subgroup had SVLT delayed-recall scores between −1.5 SD and 0. | Amyloid status was determined by 18F-florbetaben PET. Trained nuclear-medicine physicians, blinded to neuropsychological results, visually rated brain amyloid plaque load; scores 2–3 were positive and 1 was negative. | PET readers were blinded to cognitive results; EEG was not used in the PET reference classification. | EEG features associated with amyloid status; indirect screening evidence. |
| Jiao et al., 2023 | HC, MCI, AD, FTD, vascular cognitive impairment, and DLB | MCI followed Albert et al. NIA-AA criteria; probable AD followed McKhann et al.; behavioral-variant FTD and PPA followed Rascovsky/Gorno-Tempini; vascular cognitive impairment followed VASCOG; DLB followed McKeith consensus criteria. | Specialist clinical diagnoses based on established syndrome-specific criteria; a universal amyloid/tau reference standard was not required. | EEG was not reported as part of the clinical labels. | EEG-based classification models; non-AD dementias contributed only to specified differential comparisons. |
| Katayama et al., 2023 | Community-dwelling HC and MCI | MCI required performance ≥1.5 SD below age/education norms in ≥1 NCGG-FAT cognitive domain with preserved ADL; MMSE <24 indicated global impairment. HC had no domain meeting the impairment threshold. | Neurologists reviewed clinical, neuropsychological, and laboratory information using an established community-cohort algorithm; no biomarker confirmation was required. | EEG was not used in the source case definition. | EEG-based classification models. |

| **Table S17. (continued)** | | | | | |
| --- | --- | --- | --- | --- | --- |
| **Included report** | **Diagnostic groups / target condition** | **Operational diagnostic definition** | **Reference standard and adjudication** | **Independence from EEG index test** | **Contribution to this review** |
| Mazzon et al., 2018 | SCI, MCI, and cognitively healthy controls | MCI was diagnosed according to the 2011 NIA-AA criteria. SCI required subjective memory complaints for <5 years without objective impairment on neuropsychological testing. Controls had neither subjective nor objective impairment and no neurologic or psychiatric disease. | Clinical and comprehensive neuropsychological assessment; no biomarker reference standard was required for group assignment. | EEG was not used to define the diagnostic groups. | Differences in EEG features between groups. |
| Lazarou et al., 2018 | HC, SCI/SCD, MCI, and AD | Probable AD followed NINCDS-ADRDA and dementia criteria; MCI followed Petersen; SCI/SCD followed SCD-I/IWG guidance and required self-perceived memory decline within 5 years without objective impairment. Alternative vascular, psychiatric, and systemic causes were excluded. | Neuropsychiatrist/multidisciplinary clinical classification using history, neurologic examination, detailed neuropsychological testing, structural MRI, laboratory tests, and questionnaires. | EEG was not used to assign the groups. | Differences in EEG features between groups. |
| Sibilano et al., 2024 | SCD and MCI (PREVIEW cohort) | SCD followed SCD-I terminology. MCI followed NIA-AA criteria, including cognitive concern, objective impairment in ≥1 domain, preserved functional independence, and absence of dementia. | Clinical and extensive neuropsychological classification within the PREVIEW cohort; no biomarker confirmation was reported as mandatory. | EEG was not used to create the reference labels. | EEG-based classification models. |
| Sibilano et al., 2023 | HC, SCD, and MCI (PREVIEW cohort) | SCD followed SCD-I: persistent self-experienced decline from a previously normal state, unrelated to an acute event, with normal adjusted cognitive performance. MCI followed NIA-AA: concern, objective impairment, preserved functional independence, and no dementia. | Clinical and extensive neuropsychological classification in PREVIEW; HC were cognitively healthy. Biomarker confirmation was not required. | EEG was not used to assign labels. | EEG-based classification models. |

| **Table S17. (continued)** | | | | | |
| --- | --- | --- | --- | --- | --- |
| **Included report** | **Diagnostic groups / target condition** | **Operational diagnostic definition** | **Reference standard and adjudication** | **Independence from EEG index test** | **Contribution to this review** |
| Nam Heon Kim et al., 2023 | SCD or MCI, with amyloid-positive versus amyloid-negative target | SCD required persistent subjective complaint, age ≥60, education ≥6 years, memory score 0 to −1.5 SD, and other domains >−1.5 SD. MCI followed Petersen: objective memory impairment (<−1.5 SD) with preserved ADL. | Target amyloid status was based on 18F-florbetaben PET visual reads by manufacturer-trained nuclear-medicine physicians blinded to neuropsychological classification; images were dichotomized as amyloid positive/negative. | PET interpretation was blinded to neuropsychological labels; EEG was the model input and not part of the reference standard. | EEG as a screening test for brain amyloid pathology. |
| Lazarou et al., 2022 | HC, SCD, MCI, and AD, with 3-year follow-up | Probable AD followed NINCDS-ADRDA and dementia criteria; MCI followed Petersen; SCD followed IWG-2/SCD-I and required self-perceived decline within 5 years with normal examination. Competing vascular, psychiatric, systemic, and structural causes were excluded. | A neuropsychiatrist assigned baseline diagnoses using structural MRI, medical history, neuropsychological testing, and neurologic examination; follow-up cognitive status was reassessed after 3 years. | EEG was not used to establish baseline diagnoses. | Differences in EEG features and exploratory progression analyses. |
| Ganapathi et al., 2022 | SCD, MCI, and dementia (clinical syndrome) | SCD was subjective decline without MCI. MCI followed Langa and Levine, particularly objective cognitive impairment with preserved independence and no major social/occupational impairment. Dementia was a clinical syndrome category rather than an etiologic biomarker diagnosis. | Consensus of 2 board-certified dementia specialists using history, neurologic examination, MMSE/MoCA, neuropsychological assessment, and laboratory evaluation for reversible causes. | The paper explicitly states that qEEG/ERP and quantitative MRI were not used for diagnostic categorization. | EEG-based classification models. |
| Lazarou et al., 2020 | HC, SCD, MCI, and AD | Probable AD followed NINCDS-ADRDA and dementia criteria; MCI followed Petersen; SCD followed IWG-2/NIA-AA/SCD-I, with self-perceived decline within 5 years and normal general, neurologic, and neuropsychological examinations. Alternative causes were excluded. | Neuropsychiatrist classification based on medical history, clinical/neurologic examination, neuropsychological tests, structural MRI, laboratory tests, and questionnaires. | EEG was not used to assign the groups. | Differences in EEG features between groups. |
| **Table S17. (continued)** | | | | | |
| **Included report** | **Diagnostic groups / target condition** | **Operational diagnostic definition** | **Reference standard and adjudication** | **Independence from EEG index test** | **Contribution to this review** |
| Gouw et al., 2017 | Amyloid-positive SCD and MCI; clinical progression | SCD and MCI followed standard criteria (Jessen et al. and Albert et al., respectively). All primary-cohort participants were amyloid positive, defined as CSF Aβ42 <640 pg/mL; an amyloid-negative comparison group used Aβ42 ≥640 pg/mL. | Multidisciplinary consensus after a standardized dementia work-up; diagnoses were re-evaluated at follow-up. Amyloid status was based primarily on CSF Aβ42 (with amyloid biomarker assessment in the cohort). | EEG readers were blinded to baseline diagnosis and follow-up status; EEG was not used for clinical progression adjudication. | Prediction of progression to MCI/dementia. |

| **Table S18. Detailed development, validation, and performance characteristics of the included EEG prediction and artificial intelligence models** | | | | | | | |
| --- | --- | --- | --- | --- | --- | --- | --- |
| **Included report** | **Model-study type** | **Target task / outcome** | **Development sample** | **Validation / test sample** | **Data-splitting unit** | **Cross-validation** | **Predictors and feature selection** |
| Huggins et al., 2021 | Development | Three-class AD vs MCI vs healthy ageing using AlexNet | 141 participants (52 AD, 37 MCI, 52 HA); 16,197 participant-epoch images derived from 340,137 channel-level scalograms. | For each of 10 random folds: 8/10 images training, 1/10 validation, 1/10 test. | Image/epoch level; participant-level separation was not reported. | 10-fold cross-validation over random image folds. | Raw 5-s EEG epochs transformed by continuous wavelet analysis into tiled RGB scalp images; end-to-end AlexNet. |
| Engedal et al., 2020 | Development and evaluation | Prediction of conversion from SCD/MCI to dementia over approximately 5 years | Existing DI had been developed previously in a separate clinical database; current incremental logistic models used the present cohort. | 200 participants with valid follow-up (baseline SCD 45, MCI 88, HC 67); 70 converted to dementia. | Participant level. | No resampling or nested cross-validation reported for the present evaluation. | Pre-existing qEEG statistical-pattern-recognition DI; incremental logistic models added baseline cognitive-test results. |
| Jiang et al., 2025 | Development and evaluation | Binary MCI vs normal-control screening using reduced electrode configurations | 41 participants (21 MCI, 20 NC). | Preliminary independent sample of 10 participants (5 MCI, 5 NC) recruited from another community with lower education. | Participant level. | No cross-validation reported for derivation. | Theta/alpha PSD during working-memory encoding; group-difference screening followed by forward-conditional binary logistic regression across 6 electrode configurations. |

| **Table S18. (continued)** | | | | | | | | | |
| --- | --- | --- | --- | --- | --- | --- | --- | --- | --- |
| **Included report** | **Model-study type** | **Target task / outcome** | **Development sample** | **Validation / test sample** | | **Data-splitting unit** | **Cross-validation** | | **Predictors and feature selection** |
| Seul-Kee Kim et al., 2024 | Development | Four-class SCD vs naMCI vs aMCI vs AD using resting and memory-encoding EEG | 58 participants (20 SCD, 10 naMCI, 18 aMCI, 10 AD). | | Leave-one-subject-out test predictions; no external cohort. | Participant level for LOSO; EEG epochs from the held-out participant formed the test data. | Leave-one-subject-out cross-validation. | Spectral/connectivity features; one-way ANOVA screening followed by ridge-based weighting/selection; 9 ML and 3 DL algorithms. | |
| Jiao et al., 2023 | Development | HC vs MCI, HC vs AD, and HC vs MCI vs AD classification; separate regression of clinical targets | Classification: HC 246, MCI 189, AD 330 (with additional dementia groups used for specified comparisons); 38,925 EEG epochs were generated. | | Random 80% training / 20% test “samples”; within training, 5-fold training/validation. Regression used repeated participant splits. | Not stated for classification; epoch/sample-level wording suggests participant independence was not ensured. Regression was participant level. | 5-fold within the classification training set; 10 iterations of random 80/20 participant splits for regression. | PSD, entropy, complexity, connectivity and related EEG measures; label-correlation screening and forward addition of features to maximize accuracy. | |
| Katayama et al., 2023 | Development | Binary community-based MCI discrimination | 449 participants (402 HC, 47 MCI); ROSE-generated analysis sample approximately 234 HC and 215 MCI. | | Internal resampling only; no independent test cohort. | Participant level. | Monte Carlo 5-fold cross-validation, 100 iterations. | Cognitive tests, eLORETA-ICA network activity, confounders, and MRI; significant networks were z-standardized and combined using cluster-derived weights; SVM and logistic models. | |
| **Table S18. (continued)** | | | | | | | | | |
| **Included report** | **Model-study type** | **Target task / outcome** | **Development sample** | **Validation / test sample** | | **Data-splitting unit** | **Cross-validation** | | **Predictors and feature selection** |
| Sibilano et al., 2024 | Development | Binary SCD vs MCI Transformer classification | 101 PREVIEW participants (56 SCD, 45 MCI); after the test holdout, 80 participants (43 SCD, 37 MCI) remained for training/validation. | | Stratified participant-level 20% holdout test set; 5 validation folds within the remaining participants. | Participant level for train/validation/test; epochs inherited participant labels. | Stratified 5-fold cross-validation in the 80% development set. | End-to-end Transformer/self-attention using 10-, 30-, or 60-s epochs; model configuration, patch size, depth, and attention heads explored. | |
| Sibilano et al., 2023 | Development | SCD vs MCI and HC vs SCD vs MCI classification using a Transformer | 118 participants (17 HC, 56 SCD, 45 MCI). | | Each LOSO fold held out one participant; no external cohort. | Participant level; all epochs from the held-out participant were used only for testing in that fold. | Leave-one-subject-out cross-validation. | End-to-end Transformer applied to all-band and delta/theta/alpha/beta signals; no hand-crafted feature selection. | |

| **Table S18. (continued)** | | | | | | | | | |
| --- | --- | --- | --- | --- | --- | --- | --- | --- | --- |
| **Included report** | **Model-study type** | **Target task / outcome** | **Development sample** | **Validation / test sample** | | **Data-splitting unit** | **Cross-validation** | | **Predictors and feature selection** |
| Nam Heon Kim et al., 2023 | Development | Amyloid PET positivity in SCD/MCI using QEEG machine learning | 235 of 311 participants after random exclusion of 25% for testing; multicenter SCD/MCI cohort. | | 76-participant independent holdout (13 SCD Aβ+, 24 SCD Aβ−, 20 MCI Aβ+, 19 MCI Aβ−). | Participant level for the holdout; positive-class training recordings were split into halves for augmentation. | 5-fold cross-validation in model development. | Age/sex-standardized spectral and imaginary-coherence features; 6 feature-selection methods combined with 8 algorithms (48 candidate models); features associated with cognitive status were excluded. | |
| Ganapathi et al., 2022 | Development | Multinomial SCD vs MCI vs dementia classification using qEEG/ERP, with/without volumetric MRI | qEEG/ERP model: 161 participants (69 SCD, 53 MCI, 39 dementia); multimodal model: 111 participants. | | Leave-one-out predictions; no external cohort. | Participant level. | Leave-one-out cross-validation. | qEEG/ERP and MRI variables; univariable screening (p<.10) followed by stepwise multinomial logistic regression, adjusted for demographics. | |

| **Table S19** | | | | | | |
| --- | --- | --- | --- | --- | --- | --- |
| **Included report** | **Hyperparameter tuning** | **Class-imbalance handling** | **Calibration** | **External validation** | **Key out-of-sample performance** | **Possible data leakage / main interpretive limitation** |
| Huggins et al., 2021 | Manual sequential optimization of optimizer, learning-rate schedule, batch size, validation patience, and related settings using accuracy/validation performance. | Class proportions were maintained in splits; no other method reported. | Not reported. | No. | Mean three-class test accuracy 98.9% (SD 0.4%). | High leakage concern: epochs/images from the same participant could enter training and test folds; the very large number of correlated images does not replace participant-level validation. |
| Engedal et al., 2020 | Not applicable for fixed DI; model-selection details for incremental regressions were limited. | No specific method reported. | Not reported. | The pre-existing DI was evaluated in a new longitudinal cohort, but the operating cutoff and incremental models were fitted/evaluated in the same cohort. | DI AUC 0.78 (95% CI 0.70–0.85), sensitivity 71%, specificity 68%–69%, accuracy 69%. | The DI cutoff was selected in the evaluation cohort to obtain approximately 70% sensitivity; new incremental models had no separate validation. |
| Jiang et al., 2025 | Not applicable beyond regression/model/configuration selection; no formal tuning procedure reported. | No specific method reported. | Not reported. | Yes, but extremely small (n=10) and only one configuration was reported in the external sample. | Derivation AUCs: 0.683–0.830; sensitivities 0.794–0.962; specificities 0.376–0.602. External OCL4: AUC 0.771, sensitivity 0.918, specificity 0.610. | Feature/electrode screening and apparent derivation performance used the same small sample; external precision is very low and confidence intervals were not reported. |

| **Table S19 (continued)** | | | | | | |
| --- | --- | --- | --- | --- | --- | --- |
| **Included report** | **Hyperparameter tuning** | **Class-imbalance handling** | **Calibration** | **External validation** | **Key out-of-sample performance** | **Possible data leakage / main interpretive limitation** |
| Seul-Kee Kim et al., 2024 | DL early stopping after 5 epochs without improvement; other algorithm-specific tuning was incompletely reported. | Not reported. | Not reported. | No. | Best encoding-state cKNN: accuracy 93.10%, F1 92.78%, κ=0.816; class-specific AUCs 0.962, 0.890, 0.947, and 1.000. | Feature selection was not clearly nested within each LOSO fold, creating possible information leakage; very small and imbalanced classes. |
| Jiao et al., 2023 | Feature-set size chosen to maximize classification accuracy; other hyperparameter tuning not fully reported. | Not reported. | Not reported. | No. | LDA: HC vs MCI accuracy 79.8%, F1 80.9%; HC vs AD accuracy approximately 85.8%; 3-class accuracy approximately 70.2%. | For classification, the split unit is unclear and epoch-level leakage is possible; feature selection/numbers of features were optimized on the same data pipeline. |
| Katayama et al., 2023 | Caret-based model fitting; the exact hyperparameter search space was not reported. | ROSE bootstrap combination of over- and undersampling. | Not reported. | No. | Best Model 6: SVM accuracy 0.7929, sensitivity 0.7163, specificity 0.8632, AUC 0.8495; logistic accuracy 0.7795, AUC 0.8492. | Group-difference feature selection/composite construction was not clearly repeated within every resampling loop; synthetic resampling may overstate transportability. |

| **Table S19 (continued)** | | | | | | | | |
| --- | --- | --- | --- | --- | --- | --- | --- | --- |
| **Included report** | | **Hyperparameter tuning** | **Class-imbalance handling** | **Calibration** | **External validation** | **Key out-of-sample performance** | **Possible data leakage / main interpretive limitation** | |
| Sibilano et al., 2024 | Learning rate selected against validation accuracy; ablation and architecture/configuration tuning; best iteration selected by validation AUC. | | Random undersampling of majority-class epochs during training. | Not reported. | No. | Best configuration: epoch accuracy 65.4% (95% CI 63.7%–67.1%); participant accuracy 65.7%. | Although subjects were separated, multiple configurations were compared on the same holdout test set, so final performance may reflect repeated test-set use; no external cohort. The 56 SCD and 45 MCI participants appear to be the same underlying PREVIEW cohort reported by Sibilano et al., 2023 and should not be counted as independent participants. |  |
| Sibilano et al., 2023 | Adam settings were prespecified; early stopping used validation loss, but the construction of a validation subset inside each LOSO training fold was not fully described. | | Random undersampling of majority-class epochs in training. | Not reported. | No. | SCD vs MCI, delta: epoch accuracy 67.4%, AUC 0.807; participant accuracy 76.2%. Three-class: best reported micro-AUC >0.74; participant accuracy 54.2% for theta. | No external validation; the within-fold validation mechanism and isolation of preprocessing/tuning from the held-out participant were incompletely reported. The SCD/MCI sample appears to overlap completely with Sibilano et al., 2024 and should not be counted twice. |  |

| **Table S19 (continued)** | | | | | | |
| --- | --- | --- | --- | --- | --- | --- |
| **Included report** | **Hyperparameter tuning** | **Class-imbalance handling** | **Calibration** | **External validation** | **Key out-of-sample performance** | **Possible data leakage / main interpretive limitation** |
| Nam Heon Kim et al., 2023 | Algorithm/feature-set combinations and classification cutoff were searched to maximize accuracy while retaining sensitivity. | Aβ-positive training data were doubled by splitting each EEG recording into first and second halves. | Not reported. | No true external validation: the holdout was a random 25% subset of the same multicenter source population. | Holdout overall: sensitivity 90.9%, specificity 76.7%, accuracy 82.9%, AUC 0.84; SCD accuracy 81.1%; MCI accuracy 84.6%. | The holdout set was used to select final model candidates and adjust the cutoff, weakening its status as an untouched test set; extensive 48-model search increases optimism. |
| Ganapathi et al., 2022 | Stepwise selection; no separate hyperparameter procedure applicable. | No specific method reported. | Not reported. | No. | qEEG/ERP-only multinomial AUC 0.79; combined qEEG/ERP+MRI AUC 0.87. Pairwise qEEG/ERP AUCs included dementia vs MCI 0.82 and dementia vs SCD 0.87. | Pre-screening and stepwise selection were not clearly nested within each leave-one-out iteration; multimodal results used a smaller complete-case subset. |
